# GenBank mining reveals novel insights into *Rhizobium phylogeny*: Identical 16S rRNA sequences are mainly uncoupled from species designation, host plant, and geographic origin: How this search suggested the definition of a direct ‘microbial h-index’

**DOI:** 10.1371/journal.pone.0357973

**Published:** 2026-09-11

**Authors:** Rosella Muresu, Monica Rodriguez, Andrea Squartini

**Affiliations:** 1 Institute for the Animal Production System in Mediterranean Environment-National Research Council (ISPAAM CNR), Traversa La Crucca 3, Sassari, Italy; 2 Department of Agricultural Sciences, University of Sassari, Viale Italia, Sassari, Italy; 3 Interdepartmental Center for the Conservation and Valorization of Plant Biodiversity, University of Sassari, Alghero, Sassari, Italy; 4 Department of Agronomy, Food, Natural Resources, Animals and Environment, DAFNAE, University of Padova, Viale dell’Università 16, Legnaro, Padova, Italy; Free University of Bolzano: Libera Universita di Bolzano, ITALY

## Abstract

16S rDNA is the historical gold standard for bacterial identification, particularly in metabarcoding approaches reliant on sequence similarity thresholds. We analyzed 6,660 *Rhizobium* 16S rRNA gene sequences from GenBank to examine the relationship between sequence identity and three metadata: species name, host plant, and geographic origin. Using an iterative BLAST-based pipeline, we detected 116,069 pairwise matches and assessed concordance among sequences (average length 1,328 bp) sharing 100% identity. For those in which the organism name, host plant and country of isolation were present in the record, surprisingly, 66.59% of identical sequence pairs showed full discordance across all three metadata, while only 1.40% shared the same name, host, and country. The most widespread sequence, detected 371 times, was associated with over 56 different host plants across 25 countries and bore multiple species name designations. These results highlight a striking mismatch between the 16S barcode and the taxonomic, ecological, and phenotypic variability it is assumed to reflect, likely arising from the slow evolution of rRNA genes contrasted with the mobility of ecologically relevant genes via horizontal transfer on plasmids, transposons, and phages. Our findings further challenge the limitations of relying on 16S rRNA alone for fine-scale taxonomic and metadata-based inference in capturing the true functional and ecological diversity of bacteria, endorsing the critical importance of polyphasic taxonomic approaches that integrate genomic, phenotypic, and ecological data. An interesting byproduct of the analysis was to realize the possibility of treating these data as if they were ‘citations.’ The more one finds the same query sequence, the more that sequence can be considered biologically ‘cited’, i.e., re-proposed elsewhere in the world. Thus, one can also analyze the h-index of such a ranking. In our *Rhizobium* dataset, we calculated an h-index = 201, meaning the sequence ranked 201^st^ had 202 identical homologues in GenBank. Although the research effort on given species is directly connected with it, this number provides a quantitative indicator of a taxon’s sequence recurrence and distribution within public databases, independent of nomenclatural inconsistencies, offering a novel framework for assessing bacterial representation across global datasets.

## Introduction

Leguminous plants constitute approximately 20,000 species within the estimated 260,000 flowering plant species globally, representing one of the most ecologically and economically significant plant families [1]. Their remarkable success derives largely from their capacity to establish symbiotic relationships with nitrogen-fixing bacteria collectively termed rhizobia, which form specialized nodular structures on plant roots and convert atmospheric nitrogen into bioavailable forms [2–4]. This symbiosis has profound implications for crop productivity, ecosystem nitrogen cycling, and sustainable agriculture, making rhizobia among the most intensively studied bacterial groups [5, 6]. Taxonomic frameworks recognize over 400 species of rhizobia within seven families [7]. As regards the family Rhizobiaceae, recent estimates list 276 species (including both validly published and proposed ones), distributed across 38 genera [8]. The families that include rhizobia are predominantly within the classes Alphaproteobacteria and the formerly called Betaproteobacteria (now merged within Gammaproteobacteria), of the phylum Pseudomonadota, formerly Proteobacteria [9–11]. Among these, the genus Rhizobium stands as the most speciose, currently encompassing about 170 validly described species [12], which collectively encompass a vast distribution and possess the ability to interact with many diverse host plants. Based on this wide diffusion, we took the Rhizobium genus as a suitable taxon for the proof of principle of this analysis, being this taxon also currently the nitrogen-fixing legume symbiont with the highest number of known species. The taxonomic complexity of rhizobia reflects both their ecological diversity and the methodological challenges inherent in bacterial systematics, particularly due to the prevalence of horizontal gene transfer and the phenotypic plasticity characteristic of these organisms [13].

Since almost five decades, starting from the seminal work of Carl Woese [14] bacterial taxonomy has centrally relied on the nucleotide sequence of the 16S ribosomal RNA gene. This approximately 1,550 base pair-long gene encodes a structural component of the small ribosomal subunit and exhibits several properties that have established its primacy in bacterial systematics; these are: universal distribution across prokaryotes, functional constancy precluding horizontal transfer, sufficient sequence conservation to enable universal primer design, and adequate variability to discriminate among species. Taxonomic convention holds that bacterial strains sharing greater than 97.5% 16S rRNA sequence identity can be assumed to belong to the same species, though this threshold remains subject to ongoing debate and refinement [15, 16]. It is widely recognized that while 16S rRNA gene sequencing remains the gold standard for genus-level assignments, it possesses inherent limitations when resolving species-level relationships [17]. This drawback is particularly pronounced in high-throughput studies utilizing short-read amplicon sequencing of isolated hypervariable regions (e.g., V3–V4), which struggle to capture sufficient nucleotide diversity to distinguish closely related taxa reliably [18]. Our empirical findings expanding on 100% full-identity sequence pairs in *Rhizobium* strongly mirror these computational limits.

Next-generation sequencing metabarcoding studies routinely assign species-level identifications based on alignment of short amplicon sequences, typically spanning the hypervariable V3-V4 region of approximately 430 base pairs, to reference databases whose recommended format of analysis has shifted in time from the operational taxonomic units or OTUs to the amplicon sequence variants or ASVs [19, 20].

The fundamental assumption underlying these practices is that 16S rRNA sequence similarity correlates meaningfully with overall genomic relatedness, ecological function, and phenotypic characteristics. However, accumulating evidence suggests this assumption may be problematic, particularly for organisms exhibiting high rates of horizontal gene transfer or those whose ecological functions are encoded on mobile genetic elements [21]. The genomic architecture of rhizobia exemplifies these complexities, with essential symbiotic genes typically residing on large plasmids or symbiotic islands that can be transferred horizontally among divergent lineages [22, 23]. This genetic organization enables rapid acquisition of symbiotic capabilities and adaptation to novel host plants, potentially decoupling phylogenetic relationships inferred from chromosomal markers like 16S rRNA from ecologically relevant traits [10].

In response to the general challenges about 16S rDNA informativity, Parks and colleagues [11]. introduced a new phylogenetic framework based on the concatenation of 120 universally conserved proteins. This approach led to the creation of a comprehensive database, which has since expanded to include over 901.341 genomes. Within this curated classification, taxonomic groups represent monophyletic lineages of comparable phylogenetic depth, achieved by normalizing for lineage-specific evolutionary rates. This work established the Genome Taxonomy Database (GTDB), a genome-based taxonomic system that uses concatenated protein phylogenies and relative evolutionary divergence (RED) metrics to provide a standardized classification for bacterial genomes [11]. Based on such data, novel strategies have also been proposed to infer the actual recurrence proficiency of each lineage [24]. Previously, other methods to extract hidden layers of information from the metabarcoding outputs [25] or via keyword-based genetic database mining [26] had been devised.

Despite widespread recognition of the theoretical limitations of a taxonomy over-reliant on the small subunit ribosomal RNA, comprehensive empirical assessments of how well 16S rRNA sequence identity predicts ecological and taxonomic concordance remain surprisingly scarce. The exponential growth of publicly accessible sequence databases, particularly GenBank maintained by the National Center for Biotechnology Information, continues to provide unprecedented opportunities to examine these relationships on a large scale [27]. GenBank currently contains over 61 million 16S rRNA sequences accompanied by variable amounts of metadata describing the organisms from which they were derived, including taxonomic designations, isolation sources, host associations, and geographic origins [28]. Systematic mining of these data can reveal patterns that might not be apparent from focused taxonomic studies of limited isolated strain collections or individual metagenomic studies.

As mentioned, the mainstream methodology to generate results in microbiome analyses is by automated pipelines that align sequencing reads to databases and assign taxa names based on the percentage of nucleotide identities. When in such a process the BLAST algorithm yields sequences which are 100% identical to the searched query, one can suppose that, besides belonging supposedly to the same species, the organism being investigated and that of its matching subject record sequence should share a closer kinship between each other than with ones that have, e.g., just 97.6%, i.e., a situation where they are assigned to the same species but appear to represent two very diverged strains.

Under such premises, having chosen the *Rhizobium* genus as a suitable case to probe the system, the question we addressed was: should one expect as more likely that, when 16S sequences share 100% identity, they: a) were recorded with the same taxonomic name; b) were isolated from closer rather than distant sites; c) were isolated from nodules of the same host plant?

In practice, we hypothesized that, if 16S rRNA sequence identity genuinely reflects recent common ancestry and close evolutionary relationships, then Rhizobium sequences sharing perfect identity, a nearly full-length size of the gene and a complete query coverage overlap, should exhibit elevated concordance in taxonomic nomenclature, in host plant associations, and in geographic distributions, in comparison to randomly selected sequence pairs of the same organism genus. To test these predictions, we conducted a comprehensive analysis of Rhizobium 16S rRNA sequences in GenBank, selected as an ideal proof-of-principle taxon given its status as the most species-rich rhizobial genus with extensive representation in sequence databases and well-documented ecological diversity. Our analytical approach involved systematic BLAST searches of all qualifying Rhizobium 16S sequences against the complete GenBank database, identification of sequences sharing 100% identity and total overlap between query and subject, followed by quantitative assessment of metadata concordance patterns. Additionally, as a byproduct that stemmed out from the analysis, we observed the suitability of a novel metric for quantifying bacterial diversity and a given taxonomically-linked sequence representation and recurrence by adapting concepts from bibliometrics, specifically the h-index used to evaluate scientific productivity and impact [29]. The use of the real h-index, i.e., the one generated by published articles, has been proposed to be used also for bacterial pathogens since it constitutes, indirectly, a measure of their prevalence and spread [30]. In our case, the concept is brought to the actual microbial cell level, as the measured entity is not a human paper quoting a species, but a real bacterial DNA sequence.

The present study represents, to our knowledge, the first systematic large-scale evaluation of the relationship between perfect 16S rRNA sequence identity and concordance in taxonomic, ecological, and biogeographic metadata among rhizobia.

## Materials and methods

**Fig 1:**
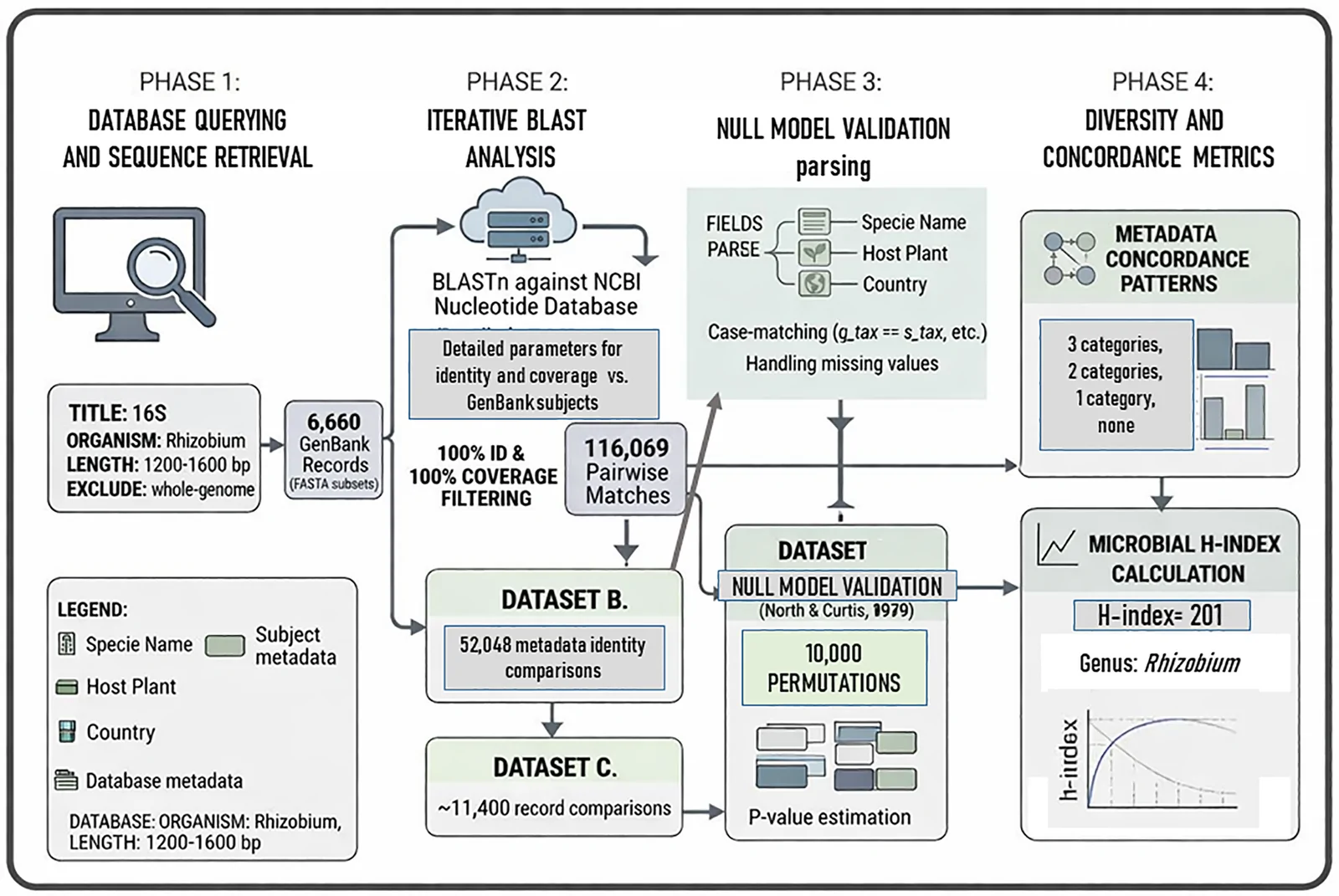
Technical workflow of the analysis.

The analytical workflow employed in this study consisted of five major phases (Fig 1): database querying and sequence retrieval, iterative BLAST analysis, metadata extraction and parsing, statistical analysis of concordance patterns, and calculation of diversity metrics. All computational analyses were performed using a combination of R statistical software version 4.0 with custom scripts, Microsoft Excel for data manipulation and filtering, and command-line BLAST tools provided by the National Center for Biotechnology Information [31]. Database querying was conducted through the BLAST+ 2.5.0 program, downloaded from the NCBI FTP site, which automatically fetches sequences from the NCBI database. A batch file was written to retrieve high-quality 16S ribosomal RNA gene sequences from organisms classified within the genus *Rhizobium* while excluding whole-genome sequences (Supporting Information S1 File, Computational workflow and Reproducibility notes). The specific query string employed was:

### 16S[Title] AND Rhizobium[Organism] AND 1200:1600[Sequence Length] NOT genome

This search strategy required that sequence records contain the term 16S in their title field, ensuring retrieval of sequences explicitly identified as 16S rRNA genes rather than other genetic elements. The organism field restriction to Rhizobium limited results to sequences from bacteria classified within this genus according to NCBI taxonomic databases at the time of deposition. The sequence length constraint of 1200–1600 base pairs was implemented to exclude partial sequences that might yield unreliable BLAST results while accommodating natural variation in 16S gene length among different bacterial lineages [32]. This initial query, executed in late 2023, retrieved a total of 6,660 GenBank records meeting all specified criteria.

All retrieved sequences were downloaded in FASTA format, the standard text-based format for representing nucleotide sequences. Given the computational constraints of performing BLAST searches with thousands of query sequences simultaneously, the complete dataset was partitioned into subsets of 20 queries each, generating 330 separate BLAST output files. Each subset was subjected to BLAST analysis against the complete NCBI nucleotide database using the blastn algorithm optimized for highly similar sequences [33]. BLAST searches were configured with default parameters for nucleotide-nucleotide comparison, with the critical exception that we retained all matches meeting our stringent filtering criteria rather than limiting to a fixed number of top hits. Results from each BLAST run were saved in tabular format and imported into Microsoft Excel for filtering and analysis. The second BLAST step was performed against the full NCBI nucleotide database, so it did not simply re-query the initial retrieval set. The ‘self matches’ were recognized and excluded from the counts by the presence of the identical accession number.

Critical filtering was applied on the global unlabeled dataset (comprising 116,069 pairwise matches) based on two essential parameters to identify sequences sharing complete identity across their entire length: Query Coverage equal to 100% and Sequence Identity equal to 100%. The combination of these two filters at 100% stringency identifies sequence pairs that are completely identical across the full length of the query sequence, representing the highest possible degree of sequence similarity and presumably indicating very recent common ancestry and relationships.

### Metadata extraction, cleaning and standardization

For each query sequence that identified one or more subject sequences meeting the 100% coverage and 100% identity criteria, we extracted three categories of metadata from the GenBank records: organism name, host plant species, and country of isolation. Metadata extraction required careful parsing of GenBank record headers and associated feature tables [34]. For our primary analysis of metadata concordance, we restricted attention to the subset of BLAST matches (mean length 1,328 bp) where all three metadata categories were explicitly provided and could be unambiguously parsed, reducing the dataset to 52,048 matches (dataset B). To reduce nomenclatural, formatting, and annotation inconsistencies (such as spelling variants, synonyms, and unverified denominations), organism and host plant names were standardized according to accepted binomial nomenclature, and geographic entries were harmonized into uniform country designations. For each sequence pair, we evaluated concordance across the three metadata categories and tested whether the observed patterns deviated from random expectation using a non-parametric null model implemented in R (Supporting Information S1 File). The model was based on 10,000 Monte Carlo permutations without replacement, preserving the marginal distributions of taxon, host, and country in the GenBank dataset while randomizing the query-subject pairing. For each iteration, we calculated the distribution of the eight possible concordance patterns, from three shared features to none, and derived the null mean, null standard deviation, and empirical p-values from the resulting simulations. An empirical threshold of α = 0.05 was used to reject the null hypothesis of random database matching. The same framework was applied to a more stringently curated subset (11,422 matches; mean length 1,326 bp; dataset C), in which taxonomic resolution was always unambiguously declared for both query and subject (Supporting Information file S2 File).

To provide an external taxonomic benchmark, the query and subject accessions from datasets B and C were cross-referenced against the SILVA curated 16S rRNA reference database (https://www.arb-silva.de/; release 138.2). This comparison was used to assess the consistency of NCBI taxonomic assignments with a phylogenetically curated framework and to quantify genus- and species-level concordance in both the uncurated global dataset and the high-resolution filtered dataset.

We then tabulated the frequency distribution of concordance patterns. To identify the *Rhizobium* 16S sequences that were highly recurrent in the database, we ranked all query sequences (dataset of 116,069 matches) in descending order based on the number of perfect matches they identified in the GenBank database. To develop a novel metric for quantifying overall diversity and individual duplication and persistence proficiency at the genus level, we adapted the concept of the h-index from science bibliometrics [29]. Specifically, a bacterial taxon is assigned an h-index of n if it features n distinct 16S identical sequences that each appear at least n times in the database. To quantify sequence recurrence at the database level, we also adapted the bibliometric h-index to the ranking of perfect 16S matches in dataset B and evaluated its associated metadata concordance in dataset C, in order to test whether highly recurrent sequences were also characterized by coherent taxonomic, host, and geographic annotation. All statistical summaries were calculated using standard functions in Microsoft Excel and verified using R statistical software.

A generative AI assistant (Perplexity AI, version Pro featuring the integrated GPT‑5.1 OpenAI module, San Francisco, CA, USA) was employed to support the editorial preparation of the manuscript. Its use was restricted to language-related tasks (grammar correction, rephrasing for clarity, and streamlining of existing text), and no sections containing data analysis, results, or scientific interpretation were originally written by the tool. All outputs were manually checked, edited, and verified by the authors, who take full responsibility for the content. The use of AI tools does not affect the reproducibility of the study, which relies solely on the described computational pipeline and publicly available data.

## Results

The starting GenBank query successfully retrieved 6,660 records representing independent sequence depositions spanning multiple decades of rhizobial research. Iterative BLAST analysis of these query sequences, employing stringent filtering criteria requiring both 100% query coverage and 100% sequence identity, generated a total of 116,069 qualifying matches representing sequence pairs that were perfectly identical across their entire aligned length. Among the 6,660 query sequences analyzed, 56.2% successfully identified at least one additional sequence in GenBank sharing 100% identity, while the remaining 43.8% were still unique (singletons), having no perfect matches in the database yet. In essence, these proportions show that for more than half of the *Rhizobium* sequences, there are already one or more identical cases that have been deposited in the database. In other words, our knowledge coverage for the *Rhizobium* denomination could be judged slightly over halfway.

For each of the 6,660 records retrieved and subsequently used in turn as queries subjected individually to the BLAST procedure against the whole GenBank, a list of matches was obtained. All these pools of identical sequences picked by each of the 6,660 queries were subsequently ranked numerically, revealing the shape of the distribution of such a phenomenon. That means, at one end, sequences that were widespread/highly occurring identically and at the other, sequences that resulted rare/unique. This way, the ranking from the most to the least successful strains of *Rhizobium* was obtained by ordering all the sequences in descending abundance by the number of matching records. This datum in practice reports how much each of those 16S sequences is represented in the current scientific knowledge.

The score thus provides, for each recurring sequence, its overall ranking in terms of the number of identical occurrences found in the worldwide body of studies. A sequence-conserving diffusion of each given sequence is used as an indicator of the reproductive potentiality of the organism bearing it, which is a measure of its ecological proficiency. Reproduction, dispersion, and persistence are variables that can contribute to such an outcome, entailing the adaptability and strength of each phylotype. The likelihood of encountering repeatedly a given sequence, either in cultured isolates or in community microbiomes, either from the same study or in different unrelated analyses carried out in distant places, provides an index to gauge the performance of that individual phylotype.

**Fig 2:**
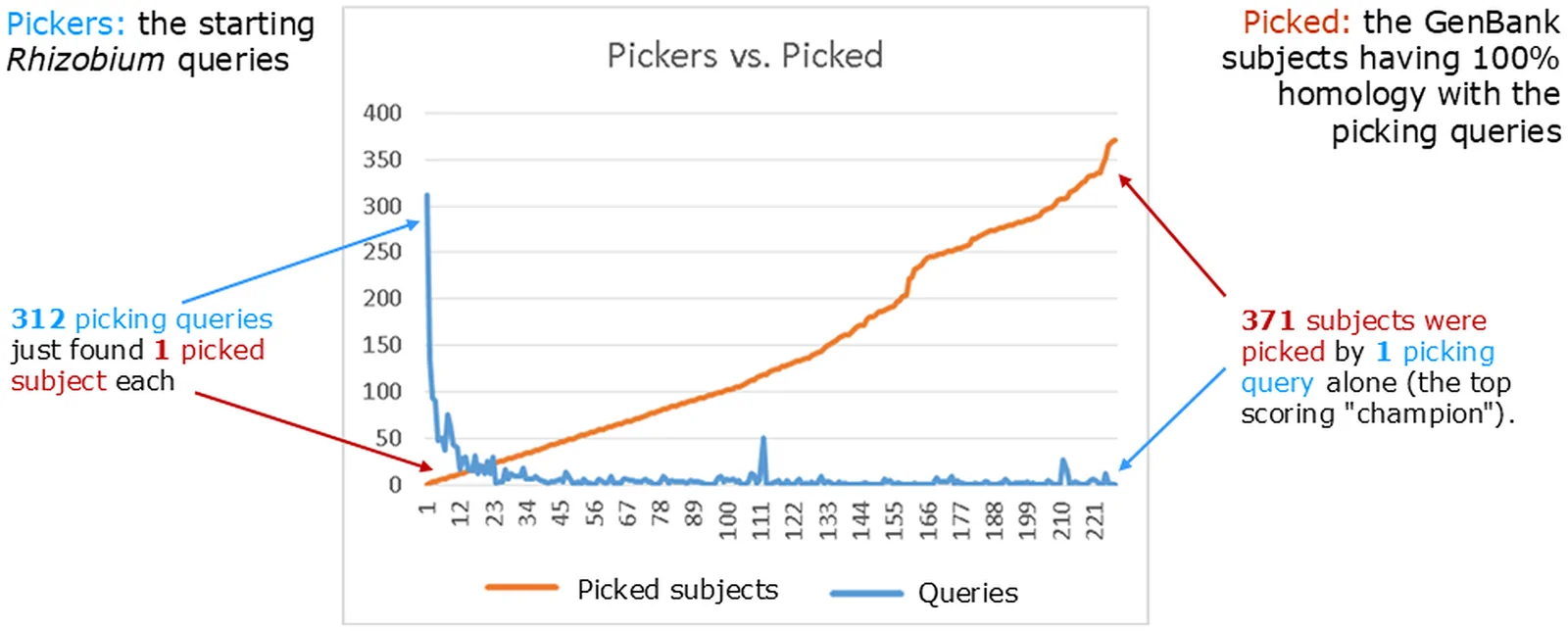
Matching efficiency for the active (picker) or passive (picked) role, that the same sequences displayed being matched across the entire database.

Within the queries that found at least one match (3743 over 6660), the most represented sequence was found 371 times (i.e., that 16S sequence was identical in 371 different subjects of the GenBank database). On the other side of the ranking, the cases that just found only one match were 312. All the other combinations lie in between. The outline of these distributional differences is shown in Fig 2.

The reason why the queries (pickers) follow an exponentially decreasing distribution while picked subjects follow a linear one is explainable by the following aspects: (a) the queries sequence length range was set to 1200–1600 bp. While sequence identity is 100%, when a shorter query (e.g., 1200 bp) matches a longer subject (e.g., 1500 bp) with 100% query coverage, the reciprocal is prevented by the 100% query coverage requisite; (b) queries were chosen with the *Rhizobium* name requisite, but when the database contains a sequence identical to the *Rhizobium* query, but not named *Rhizobium* (and therefore not belonging to the query list), one should take into account that there will be several more subjects that are picked in addition to the known queries. This further reveals that databases contain a large amount of discordant taxonomical names concealed under identical ribosomal 16S rDNA sequences.

At one extreme of the reciprocal distribution (Fig 2), 312 query sequences each identified exactly one perfect match. At the opposite extreme, a single query sequence identified an extraordinary 371 perfect matches. The distribution of match counts between these extremes followed a strongly right-skewed pattern which is also characteristic of many ecological abundance distributions [35].

Detailed analysis of the top-scoring sequence, which achieved the maximum match count of 371, provided revealing insights into the relationship between 16S identity and taxonomic, ecological, and biogeographic metadata. This ‘champion’ sequence is accessioned in GenBank under the identifier GU645019 and classified as *Rhizobium leguminosarum* strain CCNWSX1289. That strain was isolated from the host legume *Vicia tetrasperma* collected in Shaanxi, northwestern China. Despite this specific designation, the 371 perfect matches revealed a very wide heterogeneity in associated metadata. Examination of organism names demonstrated that these perfectly identical sequences bore multiple distinct species designations spanning numerous described Rhizobium species, illustrating profound inconsistencies in rhizobial nomenclature that are consistent with the remarks expressed also in prior literature and the instances for reclassification of various taxa [36, 37]. Analysis of host plant associations revealed remarkable diversity, with over 56 distinct plant species recorded as isolation sources. Geographic analysis demonstrated cosmopolitan distribution spanning over 25 countries across five continents.

As regards under which bacterial taxonomic names had those isolates bearing an identical 16S sequence had been classified, the list is shown in Table 1.

**Table 1:** Nomenclatural variety occurring across the 371 database records having a 16S rDNA sequence 100% identical to that of *R. leguminosarum* CCNWSX1289.

Host	n. cases
*Rhizobium* sp.	209
*Rhizobium leguminosarum*	82
*Rhizobium leguminosarum* bv. *viciae*	29
*Rhizobium leguminosarum* bv. *trifolii*	20
*Rhizobium leguminosarum* bv. *phaseoli*	15
*Rhizobium anhuiense*	6
*Rhizobium indigoferae*	4
*Mesorhizobium* sp.	3
*Rhizobium genosp.*	1
*Rhizobium laguerreae*	1
*Rhizobium sophorae*	1

Checking to which hosts those 371 isolates were associated, besides the plant of isolation of the picking query (*Vicia tetrasperma*), at least 58 other legumes with specified names had in their nodules bacteria with 16S sequences identical to that (and to each other). The data are shown in Table 2.

**Table 2:** Plants whose root nodules bacterial isolates bore a 16S rDNA sequence identical to that of *R. leguminosarum* CCNWSX1289 isolated from *Vicia tetrasperma.*

Host	n. cases		n. cases
plant not specified	72	*Carmichaelia australis*	1
*Vicia villosa*	66	*Clianthus puniceus*	1
*Vicia faba*	35	*Cosmos bipinnatus*	1
*Pisum sativum*	25	*Cytisus scoparius*	1
*Phaseolus vulgaris*	18	*Desmodium microphyllum*	1
*Trifolium cherleri*	16	*Hedysarum* sp.	1
*Trifolium repens*	16	*Indigofera potaninii*	1
*Lens culinaris*	13	*Kummerowia stipulacea*	1
*Vicia disperma*	12	*Lablab purpureus*	1
*Lathyrus japonicus*	11	*Lespedeza cuneata*	1
*Lathyrus cicera*	8	*Macroptilium atropurpureum*	1
*Trifolium rubens*	8	*Macrotyloma uniflorum*	1
*Vicia sativa*	8	*Mimosa pudica*	1
*Lathyrus hygrophilus*	6	*Phaseolus albescens*	1
*Trifolium glomeratum*	5	*Phaseolus vulgaris*	1
*Trifolium* sp.	4	*Robinia pseudoacacia*	1
*Glycine max*	3	*Sophora alopecuroides*	1
*Amorpha fruticosa*	2	*Sophora chathamica*	1
*Glycine soja*	2	*Sophora flavescens*	1
*Oxytropis* sp.	2	*Sophora microphylla*	1
*Vicia tibetica*	2	*Sophora prostrata*	1
*Acacia macracantha*	1	*Trifolium pratense*	1
*Acacia seyal*	1	*Trifolium semipilosum*	1
*Acacia* sp.	1	*Trifolium uniflorum*	1
*Astragalus alpinus*	1	*Tuber magnatum*	1
*Astragalus mongholicus*	1	*Vicia angustifolia*	1
*Boehmeria platanifolia*	1	*Vicia chinensis*	1
*Caragana intermedia*	1	*Vicia hirsuta*	1
*Caragana* spp.	1	*Vicia nummularia*	1

Examining then from which geographical locations were the isolates of those 371 database entries collected, the available metadata list 25 different countries covering all five continents. The data are shown in Table 3.

**Table 3:** Countries from which bacterial isolates with a 16S rDNA sequence identical to that of the *R. leguminosarum* CCNWSX1289 strain from China were isolated.

Country	n. cases
Country not specified	88
China	81
Japan	77
Spain	54
Germany	11
USA	10
Poland	8
New Zealand	7
Turkey	6
India	4
Algeria	3
Kenya	3
Pakistan	3
Mexico	2
Nepal	2
Syria	2
Argentina	1
Brazil	1
Egypt	1
France	1
Greece	1
Peru	1
South Korea	1
Taiwan	1
Tunisia	1
Ukraine	1

We have so far commented on just one single case, i.e., the most prolific among those 6,660 records. Regarding all the others and analyzing the whole dataset, the most informative way to draw meaningful information on the global situation was considered to perform the reverse type of analysis, inspecting the shared concordances of the metadata. To verify to what extent organism name, host, and country were shared among bacteria possessing an identical sequence, we first filtered the dataset to retain only those subjects for which all three metadata were available in their specific record fields. As regards the bacterial species name, we excluded those records where the denomination given was simply *Rhizobium* sp., which was featured in more than half of the cases, and that would have biased the information since several potentially different species would have been pooled in the same sorting bin, under a falsely univocal name. After these data-skimming operations, we had available 52,048 valid matches (database B) with all three variables known and unambiguous.

The question behind the analysis was: what percentage of records, besides the 100% DNA identity, would share also one feature out of those three metadata, or two of them, or all three, or none.

**Fig 3:**
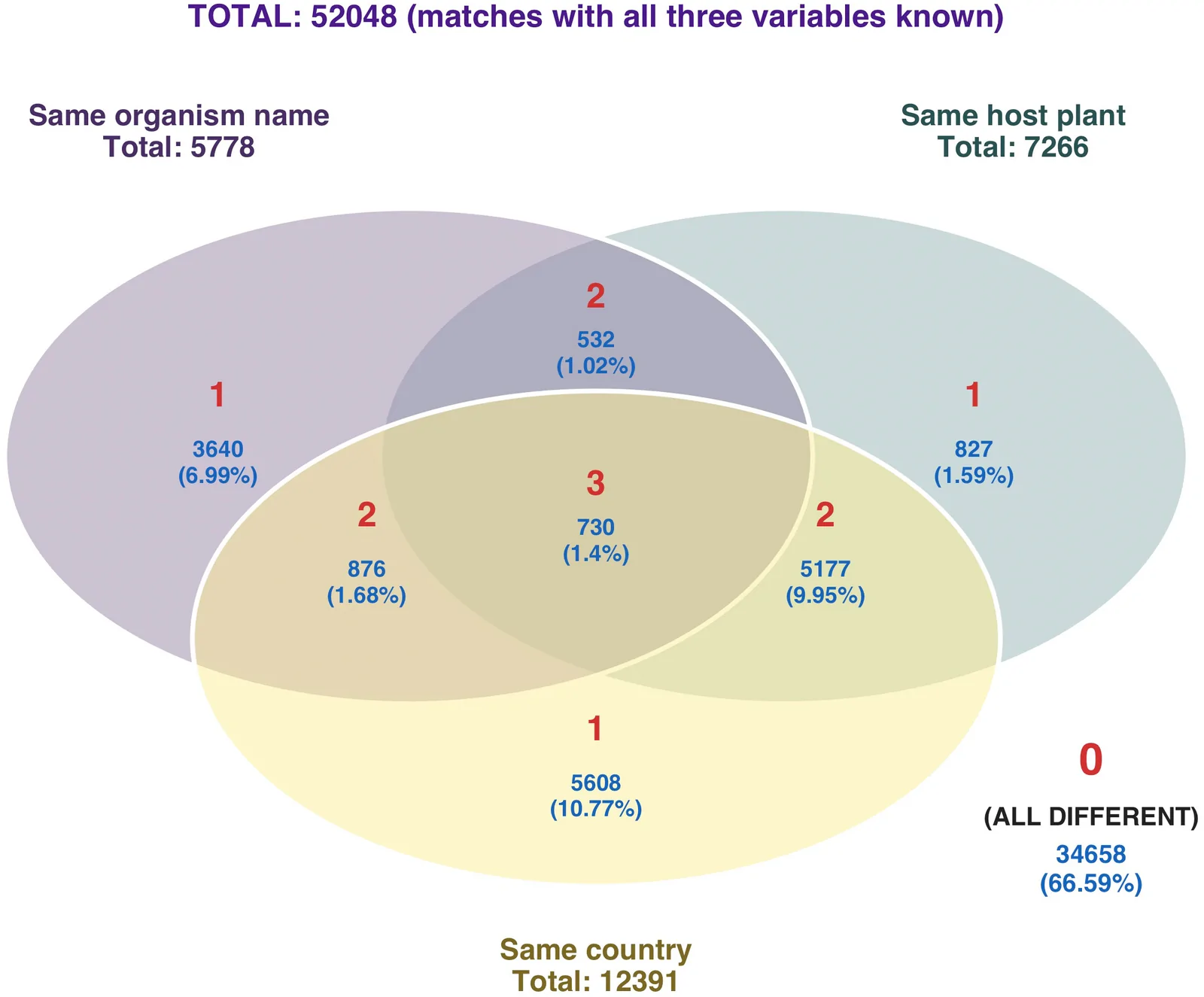
Sets of intersecting concordance in the occurrence of equal vs. different data. The condition of sharing all three conditions (3) or two (2) or only one (1), or just none (0) is shown by reporting the number of cases and the percentages of the total matches analyzed.

Such systematic analysis of metadata concordance patterns among this suitable set of sequence pairs revealed unexpected and counterintuitive patterns as illustrated in Fig 3 and recapitulated in Table 4.

**Table 4:** Distribution of shared metadata among the 52,048 sequence pairs (mean length: 1,328 bp) with 100% 16S rRNA identity and available metadata for all three categories. Statistical significance of the observed concordance patterns was assessed against a non-parametric null model using 10,000 Monte Carlo permutations without replacement.

Shared features	Concordance pattern	Number of matches	Observed %	Null Mean %	Null SD %	p-value
3	Same species AND same host AND same country	730	1.40	0.60	0.03	0.0001
2	Same species AND same host	532	1.02	0.91	0.04	0.0029
2	Same species AND same country	876	1.68	1.09	0.04	0.0001
2	Same host AND same country	5177	9.95	5.94	0.09	0.0001
1	Same host only	827	1.59	1.12	0.04	0.0001
1	Same country only	5608	10.77	8.04	0.10	0.0001
1	Same species only	3640	6.99	6.87	0.09	0.0817
0	None (all different)	34658	66.59	75.40	0.17	0.0001
	**Total**	**52048**	**100%**	–	–	–

When examining each metadata category independently, only 6.99% of the matches shared the same organism’s name, only 1.59% were isolated from the same host plant, and only 10.80% originated from the same country. Most remarkably, the single largest category by far consisted of sequence pairs sharing none of the three metadata variables, with 34,658 pairs, representing 66.59% of the total, which exhibited complete discordance despite perfect 16S sequence identity.

The test performed to verify if the observed metadata concordance differed from random expectation, showed that several multi-variable concordance classes departed significantly from the null expectation, with the “same species only” category remaining non-significant (Table 4).

We subsequently investigated the metadata concordance against a null model in the more stringently curated dataset C (11,422 matches), in which organism species names were always explicitly and unambiguously declared for both query and subject. In this subset, concordance patterns were always significantly different from the null distribution (Table 5).

**Table 5:** Distribution of shared metadata among the 11,422 sequence pairs (mean length: 1,326 bp) with 100% 16S rRNA identity and available metadata for all three categories. Statistical significance of the observed concordance patterns was assessed against a non-parametric null model using 10,000 Monte Carlo permutations without replacement.

Shared features	Concordance pattern	Number of matches	Observed %	Null Mean %	Null SD %	p-value
3	Same species AND same host AND same country	675	5.91	2.52	0.141	0.0001
2	Same species AND same host	532	4.66	4.19	0.174	0.0035
2	Same species AND same country	876	7.67	4.77	0.171	0.0001
2	Same host AND same country	181	1.58	0.793	0.0783	0.0001
1	Same host only	427	3.74	2.63	0.137	0.0001
1	Same country only	438	3.83	5.02	0.164	0.0001
1	Same species only	3625	31.74	30.4	0.281	0.0001
0	None (all different)	4668	40.87	49.7	0.256	0.0001
	**Total**	**11422**	**100%**	–	–	–

Cross-referencing the NCBI-derived accessions against SILVA returned exact matches for 960 query sequences and 965 subject sequences in dataset B, and for 585 query sequences and 395 subject sequences in dataset C. In the query set, full genus- and species-level concordance increased from 42.19% in Group B to 63.25% in Group C. In the subject set, concordance in Group C reached 85.06%, consistent with reduced annotation ambiguity after stricter curation.

Regarding the full dataset, irrespective of the presence or absence of the host or country metadata, as regards the taxonomic identities assigned to the records bearing, as genus, the *Rhizobium* name, a majority, constituting over half of the total, had the *Rhizobium* sp. definition. The remaining ones encompassed 99 different species, and the most frequent was *R. leguminosarum* (38.24%), distantly followed by *R. tropici* (6.73%) and by *R. etli* (7.92%). The whole list is shown in the Supporting Information Fig. S1 in the S2 File (Additional Data).

In total, the named host plants encountered belonged to 347 species. The most frequent was *Phaseolus vulgaris* (9.77%), followed by *Vicia faba* (7.75%), and *Vicia villosa* (5.62%). The top 50 most featured hosts (accounting cumulatively for 67.75% of the total) are listed in the Supporting Information Fig. S2 in the S2 File.

The countries in which the isolates had been collected are 75. The most featured was China (29.65%), followed by India (8.72%) and Japan (8.40%). The list is reported in the Supporting Information Fig. S3 in the S2 File.

As concerns those 43.8% of the *Rhizobium* records that had no match with any other within the database, we show their species name assignment in the Supporting Information Fig. S4 in the S2 File, after removing the *Rhizobium* sp. occurrences, which, in this subset, were above 65%. The number of different species was 68; the main one being *R. leguminosarum* (28.54%). The list of bacterial names for such ‘singletons ‘is shown in the Supporting Information Fig. S4 in the S2 File, while their 204 known plants of isolation and their 64 known countries of origin are shown in Fig. S5 and Fig. S6 S2 File, of the same file, respectively.

## Discussion

The starting key questions of this analysis, as anticipated above, were the following: should one expect as more likely that, when 16S sequences share 100% identity, implying a supposed kinship, those: a) would be recorded with the same name; b) were isolated from closer rather than distant sites; c) were isolated from the same host?

The three variables are shaped by different processes. a’) taxon name is a human choice and is related to scientific knowledge; b’) host plant is a purely biological aspect, linked to plant-microbe interactions and their co-evolution; c’) the geographic site is related to dispersal/colonization/transportation, including agricultural drive, and its context is mainly environmental.

In theory, one would assume that bacteria sharing a total identity for 16S rRNA gene, which is still officially considered the highest species-identification benchmark, should have higher probabilities to be at least enriched in sets sharing one or more of the three attributes of name, host, or site.

But, despite the above premises and considerations, upon searching in the largest repository of genetic information about the status of one of the most species-rich and worldwide diffused bacteria, we found, instead, the opposite. Up to the point that the most represented condition (66.59% of cases) turned out to be the one where all those three features were different.

A further note is worth to better clarify the geography dimension meaning and its limitations. Our dataset contains country names as reported in GenBank metadata, but not precise geographic coordinates, which are not featured by the record fields. For these reasons, we treat “country” as a coarse descriptor of sampling origin rather than as a quantitative ecological variable. We recognize that country identity alone cannot capture geographic distance or environmental similarity. However, we performed a statistical check to evaluate whether identical 16S sequences tend to cluster geographically more than expected by chance. Using the observed country frequencies in our dataset (S2 File), we computed the expected probability that two randomly drawn records could originate from the same country. This probability is 0.12, whereas the observed proportion of identical-sequence pairs from the same country is 0.11 (5,608 of 52,048). The observed value is extremely close to the expectation under random sampling, indicating that geographic origin does not structure 16S identity in this dataset. This supports our interpretation that “country” is best treated as metadata rather than as a proxy for ecological similarity.

As regards the legumes, one must also consider that, anyhow, host species identity is not a complete measure of ecological similarity, as some rhizobia can be host generalists. However, the objective of the present study was not to quantify host specificity or test for phylosymbiosis, but to evaluate the concordance of some metadata fields accompanying GenBank records. Host plant was therefore used as a standardized descriptor of isolation context rather than as a comprehensive measure of ecological similarity.

In order to test the overall reliability of our analysis, all observations were evaluated against a non-parametric null model, which allowed us to distinguish genuine concordance from patterns expected under random database composition. In dataset B, most multi-variable concordance classes departed significantly from the null distribution while the “same species only” class remained marginally non-significant, (p = 0.08), indicating that unresolved taxonomic noise still contributes to the apparent overlap among records. In dataset C, where taxonomic entries were explicitly resolved, concordance patterns departed more strongly from the null distribution, indicating that stricter taxonomic curation reduces noise and reveals a clearer biological signal.

These null-model results provide a quantitative baseline for interpreting the biological meaning of the observed discordance. In essence, they show that the profound disconnection between 16S ribosomal RNA sequence identity and concordance in taxonomic designation, host plant association, and geographic origin among Rhizobium bacteria, is not merely descriptive, but statistically structured. These data also allow us to quantitatively gauge the extent of such a phenomenon. The finding that two-thirds of sequence pairs sharing perfect 16S identity exhibit complete discordance across all three metadata categories fundamentally challenges assumptions and guidelines underlying bacterial taxonomy and metabarcoding interpretation [37].

Several interconnected factors likely contribute to the observed metadata discordance. As mentioned, current taxonomic practice relies heavily on 16S sequence similarity as a primary criterion for species delineation [19, 38, 39]. We study bacterial communities by 16S metabarcoding as an official species-defining hallmark, but it is necessary to consider some critical caveats. In first instance, its attitude, as a molecular evolutionary clock, is also tied to its nature of a passive counter and extremely slow recorder. Molecular clock estimates suggest that bacterial 16S rRNA genes evolve at extremely slow pace, with substitution rates varying across lineages but generally representing one of the most conserved genomic regions [40]. A single mutation in 16S rDNA if one considers the estimated species boundary divergence time of 166 million years, would therefore occur every 4,4 million years [41]. The extraordinarily lagging net mutation rate of those regions creates a situation where 16S sequences remain essentially frozen over evolutionary timescales during which other genomic regions undergo substantial diversification [42, 43]. In practice, the grounds we use to assign taxonomical names based on their sequence divergence are bound to the inheritance of an ancient, extremely slow-changing sequence, which, in a way, represents something similar to a person’s surname. While, on the contrary, the real ‘game-changing’ genes shaping the actual ecology of a species can enter at any stage. The penetration of plasmids, transposons, transducing phages, etc., can continuously occur and gain a chance of inheritance. However, all these genetic determinants go totally undetected by 16S metabarcoding. Therefore, the most effective genotypic and phenotypic change agents in evolutionary biology can bypass the vertical chronological timeline and just spread horizontally via mobile replicons or recombining transgenes. The resulting temporal discrepancy and the risk of missing out important genetic differences is particularly acute in rhizobia due to the genomic architecture of its symbiotic determinants [22]. The genes essential for nodulation and nitrogen fixation are predominantly located on large transmissible plasmids termed symbiotic plasmids or pSym, or on chromosomally integrated symbiotic islands [23]. Critically, symbiotic plasmids are frequently transferred horizontally among rhizobial strains through conjugation, potentially also crossing species boundaries [13, 44]. Such horizontal transfer events can instantaneously confer symbiotic capability or alter host range [10, 36]. Beyond symbiotic genes, numerous other ecologically significant traits are encoded on mobile genetic elements subject to horizontal transfer [21]. Whole-genome analyses increasingly reveal that bacterial species concepts based on single genes capture only a fraction of genomic diversity [11]. Average nucleotide identity based on whole genome comparisons can in this respect provide complementary information [45–47]. Multiphasic approaches coupling genomics with other data have been recommended for a corrected perspective [48, 49].

Rhizobial taxonomy has indeed a convoluted history characterized by numerous reclassifications [9, 50]. The disconnection between 16S identity and taxonomic nomenclature, where identical sequences bore multiple distinct species names, reflects compounding issues in rhizobial systematics [36, 51]. Different research groups have applied species names based on different criteria, creating a contradictory nomenclatural landscape. The 97.5% sequence identity threshold for species delineation based on 16S is inherently arbitrary and may not correspond to meaningful biological discontinuities [18, 52]. Some legitimately distinct species can actually share greater than 97.5% or even 100% 16S identity while differing substantially in other genomic regions [53].

Consistent with the comparison against the SILVA curated framework, these observations indicate that although 16S rRNA does not resolve fine-scale taxonomic or ecological differences, it remains a widely used marker for rapid database-based identification, broadly available, and applicable to a large number of historical and environmental accessions that lack genome sequences. Without questioning the value of genome-based taxonomy, we highlight the practical limits of relying on 16S alone when annotation quality, host information, and geographic metadata are taken into account.

The observation that identical 16S sequences show limited geographic concordance raises also questions about rhizobial biogeography and dispersal as predicted and signalled in prior literature [54, 55]. A metabarcoding sequence matching a database reference at 100% identity might derive from an organism with substantially different ecological properties [56, 57]. Recommendations have been issues on the necessity that claims of species-level identification from short metabarcoding reads should be scrutinized [18]. All these patterns reflect the extraordinarily slow evolutionary rate of ribosomal RNA genes relative to ecologically significant traits encoded on mobile genetic elements. The mosaic genome architecture of rhizobia, with critical symbiotic determinants on transmissible plasmids subject to horizontal transfer, exemplifies how phylogeny and ecology can become decoupled.

The higher importance of horizontal vs. vertical phylogeny in rhizobia and the risks of evolutionary interpretation biases when relying solely on the latter had been clearly pointed out in a key report already decades ago [58].

In essence, this comprehensive mining of *Rhizobium* 16S rRNA sequences from GenBank has revealed profound disconnects between perfect sequence identity at this phylogenetic marker and concordance in taxonomic nomenclature, host plant associations, and geographic origins. The finding that nearly two-thirds of sequence pairs sharing 100% 16S identity exhibit complete discordance across all metadata categories provides an indirect numerical quantification of the risk of biases associated particularly as regards drawing ecological inferences from the straight use of 16S microbiome metabarcoding.

This investigation made use of a major resource among our available sequence repositories, at the same time probing the high power of its content as well as some critical sides. It is in this respect worth commenting that, several known structural categories of problems of the public biological databases have been listed [59], which include mislabeling with an incorrect Linnaean affiliation, sequencing errors including chimeras sequence conflicts, for the same biological entity being represented by inconsistent sequences in different records, taxonomic conflicts, where the same accession is reclassified across releases, low resolution, ambiguous placeholders as “sp.,” missing taxa, when no reliable reference has been deposited; intraspecific variation exceeding the heuristic species threshold.

### The concept of a direct microbial H-Index

**Fig 4:**
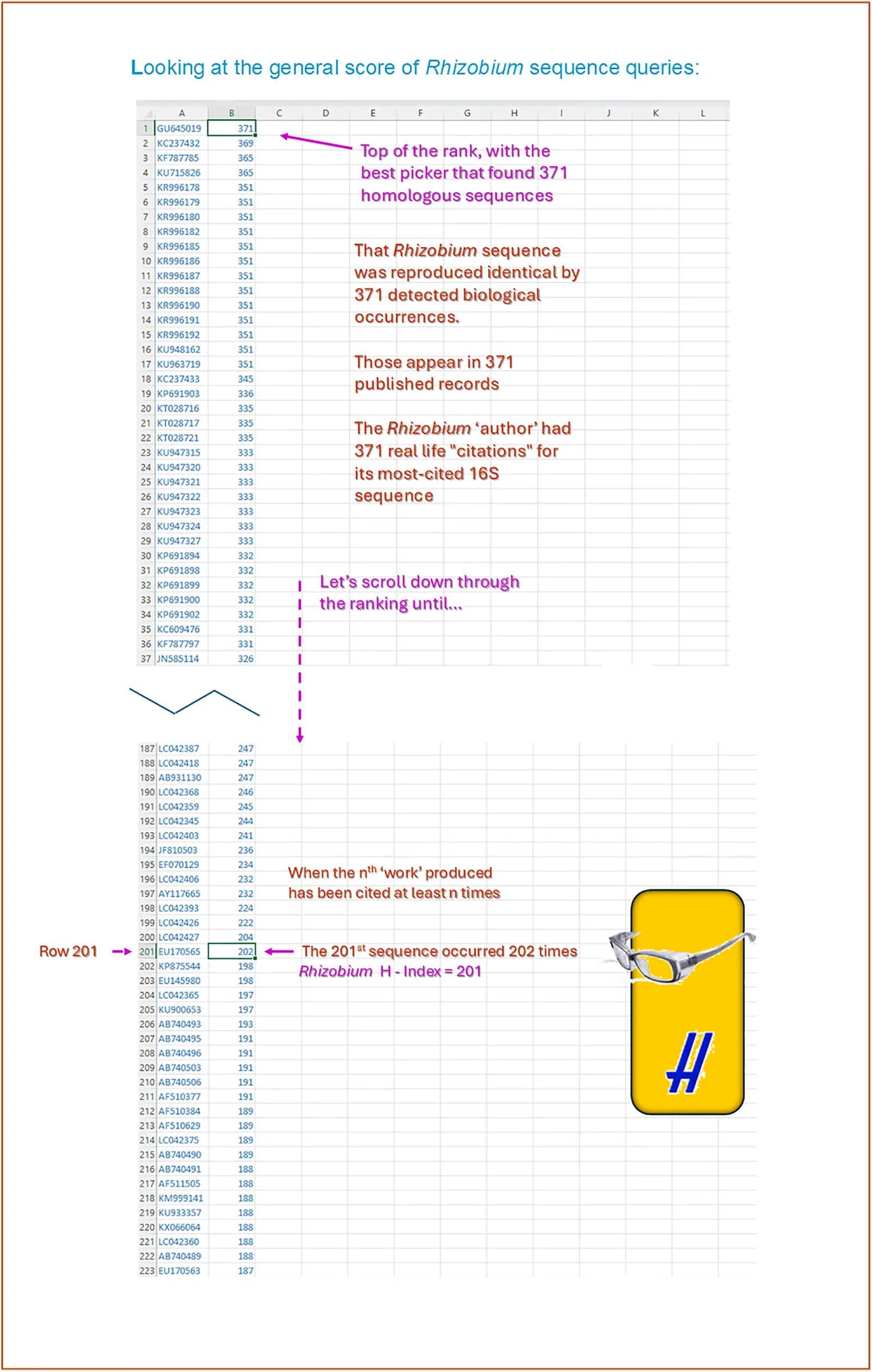
Concept illustration and calculation of the direct ‘microbial h-index’.

The idea stems actually as a by-product of the analysis presented here for the *Rhizobium* genus. The analogy with human scientific productivity metrics is based on the common aspect of the diffusion and reproduction of a product. As in the system rewarding and ruling our career, we adapted (not without a growing criticism) our selection processes to the *Publish or Perish* tenet, in nature, the survival and affirmation of individuals and species is set by their reproduction and resistance values. The ‘*publish (yourself) or perish’* is therefore embodied by the Darwinian replicator par excellence, i.e., the DNA. Under that constraint, an objective life goal is measurable by this quantitative type of metric, and our species identifier is currently still its name. Thus, for any taxon rank (phylum, genus, species, etc.), one can calculate the productive success (reproduction, persistence, diffusion) expressed by the abundance of that taxon’s corresponding set of identical ribosomal genotypes, for which our referential periscope is deposited in the continuously growing public database. How to extract such an index from the datasets is explained and illustrated in Fig 4.

By choosing the genus of interest (in the present example *Rhizobium*). and having applied the selection criteria to retrieve the GenBank records of desired length range, once the matching alignment is performed and the number of qualifying records (100% shared nucleotide identity and 100% query length coverage) is obtained, the queries are sorted in descending numerical order in terms of matching subjects and the top-scoring query is identified (in our case the one having GenBank code GU645019 and corresponding to the above mentioned strain CCNWSX1289).

Such a strain, among the members of the database classified under the *Rhizobium* genus name, has a 16S rRNA gene that, in terms of nucleotide homology, is found to be identical to 371 sequences. This means that such a sequence has been reproduced identically by 371 biological occurrences detected so far. Those appear in 371 published records. In this sense, the *Rhizobium* ‘author’ had 371 real-life ‘citations’ for its most-cited 16S sequence. By scrolling down through the ranking until one finds the equating point where the ordinal number of the series matches (or overtakes) the number of matching sequences, the index is found. This, in analogy with our way of computing an author’s production value, corresponds to saying: when the n^th^ ‘work’ produced has been cited at least n times. In this case, it occurred at row 201, indicating that the 201^st^ most abundant 16S sequence was found 202 times, which yields: *Rhizobium* h-index = 201.

While the traditional human bibliometric h-index itself has been proposed for an alternative use, i.e., to serve also as a proxy for pathogen species incidence [30], in that case, the link to microbes is indirect as it relates to species names dealt with in the published articles. In the present case, instead, we define an index based on an actual objectual entity, the DNA sequence, physically possessed by the bacterial cell, and, moreover, often independent from man-given taxonomical names, as the results of this work have unraveled.

Nevertheless, one must be aware that caution has to be used to avoid overinterpreting the possible biological significance of such metrics as a meaningful ecological or evolutionary probe. To clarify the limitations of this exploratory concept and state its boundaries of use, we can comment that

in essence; 1) in spite of the human-applied labels of taxonomical attribution, that characterize the database records, this index is of pure physical nature (occurrence of DNA bases), thus being totally independent of the possible nomenclatural inconsistencies; (2) its significance as a taxon’s ecological prevalence and achieved genetic diversity, is at the same time linked also to the amount of scientific investigation effort devoted to the organisms and habitats which can feature the occurrence of that DNA sequence. This aspect can call for a normalization (e.g., dividing by the number of database records of the taxon of choice), in case one would aim at comparing the indices of different organisms.

## Conclusions

Results demonstrate that even perfect 16S identity provides minimal information about organism name, host association, or geographic origin. These findings support growing calls for polyphasic taxonomic approaches integrating genomic, phenotypic, and ecological data.

In metabarcoding studies, researchers routinely assign taxonomic identifications to environmental sequences based on similarity to reference databases. Our findings indirectly provide a quantitative measure of the extent of misalignment between ribosomal metabarcoding data and actual biological relatedness. Data stress the concept that such assignments could be either uninformative or even misleading, particularly for organisms where ecological functions are encoded on mobile elements.

These evidence imply that claims of species-level resolution from 16S metabarcoding should be tempered with caveats about limited ecological information. Findings support growing recognition that polyphasic approaches integrating genomic, phenotypic, and ecological data are essential for meaningful bacterial systematics in the genomic era.

Finally, the bacterial h-index metric, which can be applied to any rank level and taxon, provides a novel framework for quantifying taxonomic diversity spread and a database-level metric of sequence recurrence and representation of microbial lineages.

## Supporting information

S1 File Computational workflow and Reproducibility notes. Supporting Information file S1.
(DOCX)

S2 File Additional Data. Supporting Information file S2.
(DOCX)
