# Supplementary material for: GenBank mining reveals novel insights into Rhizobium phylogeny: Identical 16S rRNA sequences are mainly uncoupled from species designation, host plant, and geographic origin: How this search suggested the definition of a direct ‘microbial h-index’
Source: PLoS One. 2026 Sep 11;21(9):e0357973. doi: 10.1371/journal.pone.0357973 (PMC13567781; doi:10.1371/journal.pone.0357973)
Supplement: Supplementary file 1 [file pone.0357973.s001.docx]

**Supporting information File S1: Computational workflow and Reproducibility notes**

This supplement documents the computational workflow used to generate the query batches, BLAST outputs, and downstream metadata tables. The analysis was based on standard tools and straightforward command-line operations, so the workflow can be reproduced directly from the files and parameters listed below.

**1. Query retrieval, batch splitting and BLAST search**

The starting query set consisted of 6,660 Rhizobium 16S rRNA sequences retrieved from the NCBI nucleotide database. These query records were split, using a custom short R script, into batches of 20 sequences each and submitted to BLAST search.

Each query batch was searched against the NCBI nucleotide database using BLAST+ 2.5.0 (blastn.exe) on Windows/DOS using the command line:

blastn.exe -db nt -query x.txt -max_hsps 1 -perc_identity 100 -qcov_hsp_perc 80 -evalue 1.0 -out x.out -outfmt "7 std" -remote

where:

-db nt searches the NCBI nucleotide database,

-query x.txt specifies the query batch,

-max_hsps 1 retains only the best HSP per hit,

-perc_identity 100 keeps only 100% identical matches,

-qcov_hsp_perc 80 requires at least 80% query coverage per HSP,

-evalue 1.0 uses the default relaxed E-value threshold,

-outfmt "7 std" produces tabular BLAST output with standard fields,

-remote runs the search on NCBI servers.

The output files were saved sequentially as x.out for each query batch.

2. Retrieval of query and subject annotations

For each query batch, the corresponding GenBank full records were downloaded from the NCBI nucleotide database and saved as .txt files. These files were then processed with a second R script to extract the fields needed for the final tables, including locus/sequence identifiers, organism name, isolation source, host, and country.

The same procedure was applied to the BLAST-hit subject records. Subject accession lists were assembled from the BLAST outputs, downloaded from NCBI as GenBank full records, and then parsed with an R script to obtain a tabular format.

**3. Data cleaning and table assembly**

The R scripts performed three main operations:

extraction of the relevant annotation lines from GenBank full records;

cleaning and harmonization of text fields;

splitting and merging of the parsed information into final tabular form.

The intermediate files (*.txt) were used to facilitate parsing and manual inspection. The final integrated tables were assembled in spreadsheet format and merged into the complete master table used for the analyses.

**4. Software and environment**

R was used for query splitting and metadata parsing.

Required R packages included: stringr, qdapRegex, gsubfn, splitstackshape, plyr, and dplyr.

BLAST searches were performed with BLAST+ 2.5.0.

Query retrieval and annotation downloads were performed using the NCBI nucleotide database and GenBank full-format records.

**5. Notes on reproducibility**

The workflow is modular and entirely based on standard operations: batch splitting in R, BLAST search against NCBI nt, and text parsing of GenBank full records. The scripts and batch files included in the supplement are sufficient to reconstruct the full pipeline without additional unpublished code.

**6. Script to run permutations on queries-subjects matches obtained from NCBI data**

\# =================================================================

\# REPRODUCIBLE R SCRIPT FOR NULL MODEL VALIDATION VIA COHESIVE PERMUTATIONS

\# =================================================================

\# This script executes a structured null model validation to assess the

\# significance of taxonomic, host, and geographical metadata concordance

\# within identical 16S rRNA sequence pairs.

\#

\# INPUT REQUIREMENTS:

\# A data frame (or tibble) containing the following pre-cleaned logical columns:

\#	- same_species : Binary indicator (1 = exact species match, 0 = mismatch)

\#	- same_host	: Binary indicator (1 = identical host plant, 0 = mismatch)

\#	- same_country : Binary indicator (1 = identical country, 0 = mismatch)

\#	- s_tax, s_hos, s_cou : Character vectors of Subject metadata (for null replication)

\#	- q_tax, q_hos, q_cou : Character vectors of Query metadata (for null replication)

\# =================================================================

library(tidyverse)

\# --- 1. EMPIRICAL DESCRIPTIVE STATISTICS ---

\# Computes the baseline observed frequencies for the 8 combinations of metadata

calculate_empirical_stats <- function(df) {

total_rows <- nrow(df)

cat("\n=======================================================")

cat("\n EMPIRICAL DESCRIPTIVE STATISTICS \n")

cat("=======================================================\n")

cat("Total pairwise identity matches analyzed:", total_rows, "\n\n")

empirical_frequencies <- df %>%

mutate(

combined_category = case_when(

same_species == 1 & same_host == 1 & same_country == 1 ~ "Same Org, Same Host, Same Country",

same_species == 1 & same_host == 0 & same_country == 1 ~ "Same Org, Diff Host, Same Country",

same_species == 0 & same_host == 1 & same_country == 1 ~ "Diff Org, Same Host, Same Country",

same_species == 1 & same_host == 1 & same_country == 0 ~ "Same Org, Same Host, Diff Country",

same_species == 1 & same_host == 0 & same_country == 0 ~ "Same Org, Diff Host, Diff Country",

same_species == 0 & same_host == 0 & same_country == 1 ~ "Diff Org, Diff Host, Same Country",

same_species == 0 & same_host == 0 & same_country == 0 ~ "Diff Org, Diff Host, Diff Country",

same_species == 0 & same_host == 1 & same_country == 0 ~ "Diff Org, Same Host, Diff Country",

TRUE ~ "Unclassified"

)

) %>%

group_by(combined_category) %>%

summarise(

Observed_Count = n(),

Observed_Percentage = (n() / total_rows) * 100,

.groups = 'drop'

) %>%

arrange(desc(Observed_Count))

print(empirical_frequencies)

return(empirical_frequencies)

}

\# --- 2. NULL MODEL COMPUTATION VIA MONTE CARLO PERMUTATIONS ---

\# Shuffles Subject metadata as an uncoupled, cohesive block to maintain internal

\# metadata covariance while breaking the true association with the Query sequence.

run_null_model_validation <- function(df_proc, n_permutations = 10000) {

total_rows <- nrow(df_proc)

categories_names <- c(

"Same Org, Same Host, Same Country", "Same Org, Diff Host, Same Country",

"Diff Org, Same Host, Same Country", "Same Org, Same Host, Diff Country",

"Same Org, Diff Host, Diff Country", "Diff Org, Diff Host, Same Country",

"Diff Org, Diff Host, Diff Country", "Diff Org, Same Host, Diff Country"

)

\# Extract empirical baseline references

empirical_values <- df_proc %>%

mutate(

category = case_when(

same_species == 1 & same_host == 1 & same_country == 1 ~ "Same Org, Same Host, Same Country",

same_species == 1 & same_host == 0 & same_country == 1 ~ "Same Org, Diff Host, Same Country",

same_species == 0 & same_host == 1 & same_country == 1 ~ "Diff Org, Same Host, Same Country",

same_species == 1 & same_host == 1 & same_country == 0 ~ "Same Org, Same Host, Diff Country",

same_species == 1 & same_host == 0 & same_country == 0 ~ "Same Org, Diff Host, Diff Country",

same_species == 0 & same_host == 0 & same_country == 1 ~ "Diff Org, Diff Host, Same Country",

same_species == 0 & same_host == 0 & same_country == 0 ~ "Diff Org, Diff Host, Diff Country",

same_species == 0 & same_host == 1 & same_country == 0 ~ "Diff Org, Same Host, Diff Country"

)

) %>%

group_by(category) %>%

summarise(

Observed_Count = n(),

Observed_Percentage = (n() / total_rows) * 100,

.groups = 'drop'

)

\# Pre-allocate results matrix for efficiency

results_matrix <- matrix(0, nrow = n_permutations, ncol = length(categories_names))

colnames(results_matrix) <- categories_names

cat("\nInitiating Null Model Validation (", n_permutations, "permutations)...\n")

set.seed(123) # Guarantees strict numerical reproducibility

for(i in 1:n_permutations) {

if(i %% 2500 == 0) {

cat(" Progress:", round((i / n_permutations) * 100, 0), "% completed...\n")

}

\# METHODOLOGICAL CORE: Subject row metadata are randomized together as a

\# single block to preserve biological/ecological metadata covariance.

random_order <- sample(1:total_rows, size = total_rows, replace = FALSE)

s_tax_null <- df_proc$s_tax[random_order]

s_hos_null <- df_proc$s_hos[random_order]

s_cou_null <- df_proc$s_cou[random_order]

\# Evaluate stochastic consensus on randomized pairings

ss_null <- if_else(!is.na(df_proc$q_tax) & !is.na(s_tax_null) & df_proc$q_tax == s_tax_null, 1, 0)

sh_null <- if_else(!is.na(df_proc$q_hos) & !is.na(s_hos_null) & df_proc$q_hos == s_hos_null, 1, 0)

sc_null <- if_else(!is.na(df_proc$q_cou) & !is.na(s_cou_null) & df_proc$q_cou == s_cou_null, 1, 0)

cat_null <- case_when(

ss_null == 1 & sh_null == 1 & sc_null == 1 ~ "Same Org, Same Host, Same Country",

ss_null == 1 & sh_null == 0 & sc_null == 1 ~ "Same Org, Diff Host, Same Country",

ss_null == 0 & sh_null == 1 & sc_null == 1 ~ "Diff Org, Same Host, Same Country",

ss_null == 1 & sh_null == 1 & sc_null == 0 ~ "Same Org, Same Host, Diff Country",

ss_null == 1 & sh_null == 0 & sc_null == 0 ~ "Same Org, Diff Host, Diff Country",

ss_null == 0 & sh_null == 0 & sc_null == 1 ~ "Diff Org, Diff Host, Same Country",

ss_null == 0 & sh_null == 0 & sc_null == 0 ~ "Diff Org, Diff Host, Diff Country",

ss_null == 0 & sh_null == 1 & sc_null == 0 ~ "Diff Org, Same Host, Diff Country"

)

null_counts <- table(cat_null)

for(name in categories_names) {

pct_value <- if(name %in% names(null_counts)) (null_counts[name] / total_rows) * 100 else 0

results_matrix[i, name] <- pct_value

}

}

\# Statistical synthesis & Empirical P-value estimation (North & Curtis, 1979)

null_synthesis <- as.data.frame(results_matrix) %>%

summarise(across(everything(), list(

Null_Mean = ~ mean(.),

Null_SD	= ~ sd(.)

))) %>%

pivot_longer(everything(), names_to = c("category", ".value"), names_pattern = "(.*)_(Null_Mean|Null_SD)")

final_report <- empirical_values %>%

left_join(null_synthesis, by = "category") %>%

rowwise() %>%

mutate(

category_distribution = list(results_matrix[, category]),

p_value = if_else(

Observed_Percentage > Null_Mean,

(sum(category_distribution >= Observed_Percentage) + 1) / (n_permutations + 1),

(sum(category_distribution <= Observed_Percentage) + 1) / (n_permutations + 1)

)

) %>%

select(-category_distribution) %>%

arrange(desc(Observed_Percentage))

cat("\n[!] Null Model Validation Completed.\n")

print(final_report)

return(final_report)

}

\# =================================================================

\# PIPELINE EXECUTION EXAMPLE

\# =================================================================

\# Assuming 'df_clean' is your pre-verified, standardized data frame:

\#

\# observed_stats	<- calculate_empirical_stats(df_clean)

\# validation_table <- run_null_model_validation(df_clean, n_permutations = 10000)
