# Supplementary material for: GenBank mining reveals novel insights into Rhizobium phylogeny: Identical 16S rRNA sequences are mainly uncoupled from species designation, host plant, and geographic origin: How this search suggested the definition of a direct ‘microbial h-index’
Source: PLoS One. 2026 Sep 11;21(9):e0357973. doi: 10.1371/journal.pone.0357973 (PMC13567781; doi:10.1371/journal.pone.0357973)
Supplement: Supplementary file 2 [file pone.0357973.s002.docx]

**Supporting Information file S2. Additional data**

Fig. S1. Species proportions in the whole dataset. The featured names retrieved under the query *Rhizobium* as genus are reported with their frequencies. The records reporting the generic term *Rhizobium* sp. (constituting the main entry with more than half of the cases) are not listed.

Fig. S2. Top 50 hosts over a total of 346 (accounting for 67.75%).

Fig. S3. Country distribution. The nations in which database records reported the isolation site are listed with their percentages over the total.

Fig. S4. Species proportions of ‘singles’, i.e., records named under the *Rhizobium* genus (excluding *Rhizobium* sp.) that did not meet the criterion of sharing 100 % identity with any of the other records of the *Rhizobium* dataset, remaining unique variants.

Fig. S5. Plant species of reported isolation for the cases not displaying any 100% match within the *Rhizobium* dataset (singles’). Notice the occurrence of many non-legumes, suggesting the endophytic nature of strains compliant with a *Rhizobium* 16S rDNA sequence associated with other plants.

Fig. S6. Country distribution of the unique variants (singles’) described in Fig. S3.

**Table S1.** Taxonomic concordance between NCBI and SILVA databases across databases B (no: 52,048 matches) and C (no 11,422 matches) relative to query and subject sequence metadata.

| **Taxonomic concordance category** | **Dataset B Query (n = 960)** |  | **Dataset B Subj (n = 965)** |  | **Dataset C Query (n = 585)** |  | **Dataset C Subj (n = 395)** |  |
| --- | --- | --- | --- | --- | --- | --- | --- | --- |
| Matches | no. | % | no. | % | no. | % | no. | % |
| Both concordant (genus and species match) | 405 | 42.19 | 413 | 42.80 | 370 | 63.25 | 336 | 85.06 |
| Genus concordant only | 399 | 41.56 | 406 | 42.07 | 156 | 26.67 | 0 | 0.00 |
| Both discordant | 116 | 12.08 | 86 | 8.91 | 33 | 5.64 | 0 | 0.00 |
| Species concordant only | 36 | 3.75 | 53 | 5.49 | 24 | 4.10 | 53 | 13.42 |
| Genus concordant only, species different/absent in SILVA | 4 | 0.42 | 7 | 0.73 | 2 | 0.34 | 6 | 1.52 |
| **Total** | **960** | **100.00** | **965** | **100.00** | **585** | **100.00** | **395** | **100.00** |

**Note:** Concordance was assessed by comparing NCBI taxonomic annotations with SILVA Release 138.2. Database B represents the dataset, where metadata are present for all the three variables: organism, host and country name, whereas dataset C represents the filtered high-resolution dataset where ambiguous metadata assignment were removed.
