# Supplementary material for: GenBank mining reveals novel insights into Rhizobium phylogeny: Identical 16S rRNA sequences are mainly uncoupled from species designation, host plant, and geographic origin: How this search suggested the definition of a direct ‘microbial h-index’
Source: PLoS One. 2026 Sep 11;21(9):e0357973. doi: 10.1371/journal.pone.0357973 (PMC13567781; doi:10.1371/journal.pone.0357973)
Supplement: Supplementary file 3 [file pone.0357973.s004.docx]

**Reviewer #1:** The findings of this study critically highlight the increasing call for taxonomic approaches that go beyond the 16S rRNA gene, integrating phenotypic and ecological data. The manuscript also introduces a particularly innovative approach by applying the h-index concept to “citations” of sequences, providing a useful metric to assess the ecological prevalence and global distribution of bacterial taxa.
The manuscript is well written, clear, and logically organized, with an English level in line with the journal’s standards.
The "Materials and Methods" section is detailed and easy to follow. As a minor recommendation, I suggest including a diagram or figure to illustrate the analytical workflow to improve the clarity for the readers.
The Results are clearly presented, and the interpretations in the Discussion are consistent logic. The Conclusions are well supported by the data.
Overall, given its novelty, I suggest accepting this manuscript for publication.

ANSWER: We appreciate these positive remarks about quality and novelty, as well as for endorsing publication. As suggested, we have added a workflow diagram scheme, depicting all the analytical phases of the study, including sequence retrieval, iterative BLAST analysis, metadata extraction and cleaning, statistical concordance analysis, and calculation of diversity metrics. This graphical outline now also includes all the new analyses, which have been added for the verification of the statistical significance of the results by comparing those of a null model and further checks. Given the importance that reviewers have signalled for the inclusion of these control analyses, we propose that this outline could become a new figure (New Fig.1).

**Reviewer #2:** Greetings
Very good work. But kindly edit the points below:
Major Comments
1. Lack of Robust Statistical Analysis
The study relies primarily on descriptive statistics (percentages and counts), without applying inferential statistical tests to support the conclusions.
For example, the reported high proportion of discordance among identical sequences is intriguing, but it is unclear whether this observation significantly deviates from what would be expected by chance.
The authors should:
Apply appropriate statistical tests (e.g., chi-square tests, permutation tests, or null model comparisons).
Provide confidence intervals where applicable.
Consider modeling approaches to quantify associations between sequence identity and metadata concordance.
Without this, the conclusions remain observational rather than analytically supported.

ANSWER: First, we thank the reviewer for opening the report with the positive recognition *Very good work*. We equally acknowledge the relevant comments about implementing the analysis with an appropriate independent statistical backup, and, as in part anticipated above, for this purpose we strengthened the analytical framework by introducing a non-parametric null model based on 10,000 Monte Carlo permutations without replacement, designed to preserve the marginal frequencies of taxon, host, and country metadata while randomizing only the query-subject pairing. This framework was applied to both the broad metadata-complete dataset (dataset B; 52,048 matches) and to a more stringently curated subset (dataset C; 11,422 matches), allowing us to test whether the observed concordance patterns differed from random expectation. These additions were incorporated in the Materials and Methods section under “Metadata extraction, cleaning and standardization” and the corresponding results are now reported in the Results section in the paragraphs introducing Tables 4 and 5, as well as in the opening part of the Discussion.

The repetition of the analysis on updated data led to slight modifications in the numbers and proportions, leading also to a new revised version of the prior Fig. 2

2. Reliability and Bias of GenBank Metadata
The study heavily depends on metadata extracted from GenBank records (species name, host, country), yet these fields are known to be inconsistent, incomplete, and sometimes inaccurate.
Although the authors filtered the dataset to include only entries with complete metadata, there is no critical discussion of:
Potential annotation errors
Inconsistent taxonomic assignments
Sampling and submission bias
This is a major limitation that could significantly influence the results.
The manuscript would benefit from:
A dedicated paragraph discussing metadata reliability
A sensitivity analysis or validation approach, if possible

ANSWER: We agree with the Reviewer that GenBank metadata can contain inconsistencies, incomplete annotations, and historical nomenclatural ambiguity. To address this issue, we explicitly added a metadata-cleaning and standardization step in the revised Materials and Methods, including harmonization of organism names, host plant names, and country designations, and we introduced a more conservative high-resolution subset (dataset C) in which taxonomic assignments were explicitly resolved for both query and subject. In addition, the revised Discussion now contains a dedicated interpretation of how unresolved taxonomy contributes to apparent overlap in dataset B, whereas stricter curation in dataset C reduces annotation noise and reveals a clearer biological signal.

About the inherent weaknesses of the available databases, we agree on the fact that, independently of our present purpose, different aspects need to be considered to properly frame the limits that public repositories pose to all studies that mine their content. This concept has been further remarked in the text and, in particular, by recapitulating their different structural limitations as evidenced by Keck, et al (2023). *Navigating the seven challenges of taxonomic reference databases in metabarcoding analyses.* Mol. Ecol. Res., 23, 742–755.

3. Absence of Genomic-Level Validation
A central claim of the manuscript is that 16S rRNA identity is insufficient for reflecting ecological and taxonomic relationships. While this is a recognized issue, the study does not provide any comparison with genome-based approaches.
Including discussion or comparison with:
Average Nucleotide Identity (ANI)
Genome Taxonomy Database (GTDB)
Whole-genome phylogeny
would greatly strengthen the argument and place the findings in a broader modern taxonomic context.

ANSWER: We thank the Reviewer for this constructive suggestion. Although full genome-based analyses such as ANI or whole-genome phylogeny fall outside the primary scope of the present study, which focuses on the practical use of 16S-based NCBI BLAST annotation, we added an external taxonomic comparison against the SILVA curated 16S rRNA framework (release 138.2). This comparison was not intended as a complete reclassification of the NCBI dataset, but rather as an external curated reference point to place the NCBI-based results in a broader taxonomic context and to assess whether stricter filtering improved taxonomic coherence. The corresponding text has been added in the Materials and Methods section, the Results section in the paragraph following Tables 4 and 5, and the Discussion section where the practical use and limits of 16S-based annotation are discussed.

We also cross-referenced our NCBI-derived accession pool against GTDB as an external genome-based check. This search returned only 88 RefSeq matches, indicating that genome-level coverage for the records under study is limited. Because our question concerns the taxonomic interpretation of 16S records rather than genome mining per se, we only complemented our check with SILVA for resolving species-level attribution.

4. Interpretation of Results is Sometimes Overstated
The manuscript occasionally presents conclusions in a way that may overgeneralize the findings.
While the data clearly highlight limitations of 16S-based inference at fine resolution, it is important to acknowledge that:
16S rRNA remains useful for higher-level classification (e.g., genus-level)
Its limitations are context-dependent
The conclusions should be revised to reflect a more balanced and nuanced interpretation.

ANSWER: We appreciate this observation and have revised the Discussion to present a more balanced interpretation. In particular, we now explicitly state that, although 16S rRNA does not resolve fine-scale taxonomic or ecological differences, it remains a widely used marker for rapid database-based identification and continues to be broadly applicable to the many historical and environmental accessions that lack genome sequences. We also clarified that our results do not question the value of genome-based taxonomy but rather highlight the practical limits of relying on 16S alone when annotation quality, host information, and geographic metadata are taken into account.

5. The “Microbial h-index” Concept Requires Caution
The proposed “microbial h-index” is an interesting and creative idea. However, in its current form, it appears largely conceptual and lacks biological validation.
Before presenting it as a meaningful ecological or evolutionary metric, the authors should:
Clarify its limitations
Avoid overinterpreting its biological significance
Present it more cautiously as a preliminary or exploratory concept

ANSWER: We are aligned on this caveat, and we have accordingly re-toned the definition and stated its boundaries of usefulness. We have better clarified that, in essence; 1) in spite of the human-applied labels of taxonomical attribution, that characterize the database records, this index is of pure physical nature (occurrence of DNA bases), thus being totally independent of the possible nomenclatural inconsistencies; (2) its significance as a taxon’s ecological prevalence and achieved genetic diversity, is at the same time linked also to the amount of scientific investigation effort devoted to the organisms and habitats which can feature the occurrence of that DNA sequence. This aspect can call for a normalization (e.g., dividing by the number of database records of the taxon of choice), in case one would aim at comparing the indices of different organisms.

**Reviewer #3:** ========
What are the main claims of the paper and how significant are they for the discipline?
========
The authors main claims are to use the Rhizobium genus to examine concordance of microbial species taxonomies and ecological similarities with 16S rDNA sequence. They frame their results as a “challenge [to] the increasingly weakening reliability of 16S based species identification in capturing the true functional and ecological diversity of bacteria”. They also introduce a quantitative (h-index) described as an indicator of a taxon’s ecological prevalence and genomic diversity, which is independent of nomenclatural inconsistencies.
Their proposed scientific question at the end of the introduction is: “Under such premises, having chosen the Rhizobium genus as a suitable case to probe the system, the question we addressed was: should one expect as more likely that, when 16S sequences share 100% identity (‘clonal kins’) they: a) were recorded with the same taxonomic name; b) were isolated from closer rather than distant sites; c) were isolated from nodules of the same host plant ?”
Rhizobium is an important bacterial genus with implications for One Health, crop productivity, ecosystem nitrogen cycling, and sustainable agriculture, making this genus an intensively studied and important bacterial group for human enterprise. In addition, methods which utilize already existing data in publicly available databases are important contributions to the scientific community.

ANSWER: We thank Reviewer 3 for the extensive and thoughtful critique. Several of the issues raised overlap with concerns also noted by Reviewer 2, particularly regarding the need for a formal statistical framework, the interpretation of metadata concordance, and the degree of caution required in discussing the biological meaning of the results. In the revised manuscript, we have addressed these points by proposing a statistical analysis adapted to validate our observations, clarifying the scope of the study, improving the description of the methods, and moderating several formulations in the Introduction, Results, and Discussion.

In addition, we sincerely appreciate the correct understanding of the aims, including the fact that the Reviewer has caught the proper distinction of the “biological h-index” analogy. In fact, the concept stemmed from the data of the main analysis and appeared to us as worth reporting, also because, in spite of a scenario of little concordance of the records' environmental metadata, it indeed appears as a pure number that is independent of nomenclatural inconsistencies. We have added this suggested useful refined specification to its description.

========
Do the data and analyses fully support the claims? If not, what other evidence is required?
========
The analyses do not support the author's claims or question. The paper is only qualitative in nature, and no statistical framework is employed to test the author's hypothesis. This leaves the main questions (line 124) “should one expect as more likely that, when 16S sequences share 100% identity they (a) were recorded with the same taxonomic name, (b) were isolated from closer rather than distant sites (c) were isolated from nodules of the same host plant?” unanswered. Because the authors constrain the analysis only to sequences with 100% identity, they do not have the appropriate dataset to support statistical analysis of their proposed framework. For publication, the authors would need to develop and employ such a statistical framework.

In general, throughout the paper, only descriptive qualitative analyses are reported.

ANSWER: We thank the Reviewer for this important observation. We agree that a design restricted to 100% identity matches does not represent the full continuum of 16S divergence. However, we would like to underline that the objective of our study was not to model the entire gradient of sequence similarity, but rather to test the most stringent condition under which metadata concordance might reasonably be expected to be maximal, namely complete sequence identity combined with full query coverage across the aligned region. In this sense, the 100% identity criterion was used as a deliberate boundary condition for the analysis, not as an implicit claim that sequence identity alone is sufficient to establish species equivalence or full ecological similarity.

To address the Reviewer’s concern more rigorously, and as also requested by Reviewer 2, we re-evaluated the structure of our dataset and strengthened the analytical framework in the revised manuscript. We now distinguish a global full dataset (116069 matches) from two subsets: dataset B, comprising 52,048 full-length 100% identity matches with complete metadata, and dataset C, a more stringent subset of 11,422 matches in which species-level identification is explicit and unambiguous for both query and subject. The transition from B to C derives from a conservative anti-noise filtering strategy designed to exclude uninformative or taxonomically ambiguous entries, such as environmental clones, uncharacterized isolates, and generic strain designations, for example “*Rhizobium* sp.” records lacking unequivocal species resolution, or entries such as “*Rhizobium* sp. SEMIA 6437” and “Rhizobium sp. tg1”.

Within this revised framework, we believe the statistical analysis is no longer purely descriptive. We implemented a non-parametric null model based on 10,000 Monte Carlo permutations without replacement, preserving the marginal distribution of taxon, host, and country while randomizing the query-subject association. This allowed us to test directly whether the observed concordance patterns differ from random expectation within the background composition of the GenBank dataset itself. Under this model, several concordance classes in dataset B differed significantly from the null expectation, whereas the “same species only” class remained non-significant (p = 0.0817), which we consider informative because it reveals the contribution of unresolved or noisy taxonomic annotation in the broader dataset rather than a failure of the framework. In the stricter dataset C, where ambiguous records were removed, the biological signal became clearer; for example, the fully concordant class, where species, host, and country were all shared, increased to 5.91% compared with a null expectation of 2.52% (p = 0.0001).

To respond specifically to the Reviewer’s concern about the lack of an internal baseline, we also recovered a previously discarded subset of short alignments (<500 bp) and treated it as an additional high-noise reference baseline, without including it in the manuscript. We applied the same null model to this dataset (N = 15,243), using it as a negative-control, and we observed that concordance across all three variables was lower than expected under the null model (0.05% observed vs 0.28% expected), consistent with the interpretation that shorter alignments provide a poorer and less informative background for taxonomic comparison.

| **Shared features** | **Concordance pattern** | **Number of matches** | **Observed %** | **Null Mean %** | **Null SD %** | **Empirical p-value** |
| --- | --- | --- | --- | --- | --- | --- |
| **3** | Same species AND same host AND same country | 7 | 0.05 | 0.2820 | 0.0413 | 0.0001 |
| **2** | Same species AND same host | 132 | 0.87 | 0.2040 | 0.0355 | 0.0001 |
| **2** | Same species AND same country | 43 | 0.28 | 0.0714 | 0.0211 | 0.0001 |
| **2** | Same host AND same country | 61 | 0.40 | 0.1300 | 0.0286 | 0.0001 |
| **1** | Same host only | 256 | 1.68 | 1.1000 | 0.0773 | 0.0001 |
| **1** | Same country only | 421 | 2.76 | 0.9900 | 0.0761 | 0.0001 |
| **1** | Same species only | 1102 | 7.23 | 3.4100 | 0.1320 | 0.0001 |
| **0** | None (all different) | 13221 | 86.73 | 93.8000 | 0.1750 | 0.0001 |
| **Total** | **TOTAL RECORDS** | **15243** | **100%** |  |  |  |

For these reasons, we respectfully submit that the revised study now includes an explicit and testable statistical framework, while preserving the original rationale of focusing on perfect 16S identity as the strongest possible sequence-similarity condition. We hope this clarification addresses the Reviewer’s concern that the 100% identity design was too narrow to support inference.

Conceptually, one can also consider that, scaling down from an alignment of a dataset featuring a mean length of 1328 bp to a dataset of <500, is in practice allowing the loosening of over 828 matching points as if they were now allowed to be different, as they are indirectly excluded from the 100% matching. This aspect in a way, also addresses the issue of checking the behaviour of data when testing also matches at 99% , 98% 97% etc. as the length through which stringency is applied becomes instantly much lower.

The authors seem to use subspecies mismatches as evidence of species-level discordance. This would need to be corrected.

ANSWER: Actually, the terms subspecies or subsp. are completely absent from the manuscript either as text or compiled tables reporting names which appear in the database, and we have not dealt with that rank denomination. We may imagine that the Reviewer is intending the undefined species (sp.). If that were the case, however, the issue of false positivity for corresponding names is avoided by excluding those cases from the matching counts. Such a choice equally avoids the potential false negativity aspect from affecting the calculations of concordance. This aspect of the protocol is explained in the following sentences of the manuscript:

“As regards the bacterial species name, we excluded those records where the denomination given was simply *Rhizobium* sp., which was featured in more than half of the cases, and that would have biased the information since several potentially different species would have been pooled in the same sorting bin, under a falsely univocal name. After these data-skimming operations, we had available 52,048 valid matches with all three variables known and unambiguous.”

The authors propose that a measure of ecological similarity between two microbes is the host species identity from which they are isolated, but it is unclear if this is an appropriate indicator of microbial ecological similarity for the system in question. Some species are known to be generalists and might exist in multiple hosts. No statistical analysis was done to look at host relatedness and microbial relatedness for evidence of phylosymbiosis.

ANSWER: We thank the reviewer for giving us the opportunity to add a clarification to address this important aspect. The latest available report on the host range breadth, exploring 109 legume species, is in this sense very useful to describe the knowledge about generalist vs. specialist legume-nodulating bacteria (Rojas-Jimenez, et al. (2026). *Exploring symbiotic legume-rhizobia relationships across tropical species*. World Journal of Microbiology and Biotechnology, 42(2), 75. As also emerged from prior literature, the broad host range generalist nitrogen-fixing symbionts are essentially represented by members of the *Bradyrhizobium* genus, and to a lesser extent by *Mesorhizobium*. As regards instead other genera, including the genus *Rhizobium,* as well as *Paraburkholderia*, *Curpiavidus* etc., their typical number of compatible hosts, as confirmed by those authors, is limited to 1-2 species. In our survey we have chosen a non-generalist, narrow-host-range taxon. Being the average number of hosts for a *Rhizobium* in the low end of the scale (1-2) the fact that we observe record labels that list as many as 58 different plant species to records sharing a 100% identical *Rhizobium* 16S sequence, suggests that the observed data therefore fall well beyond the intervals of possible true biological host range constraints of this genus.

We also need to point out that host range is not a species-level constant either but varies at the strain/biovar/symbiovar level.

A correlated consequence is that host identity alone does not predict microbial phylogeny or ecological similarity, being in this sense an imperfect metadatum field, as far as ecological prediction is concerned.

Being our question: *Given identical 16S sequences, how often do we observe the same recorded host?* This does not equate to a question as: *Are the hosts phylogenetically related?*

Our work is in fact about metadata concordance, not about host-specificity evolution, and we did not claim that a different host would indicate a different ecological niche either. We only measured whether the recorded host metadata coincide.

We have now clarified in the text that our paper is not about co-evolution, whereas it is really a verification about the information content of a database descriptor.

In summary, we appreciate this observation. We agree that host species identity is not a complete measure of ecological similarity and that some rhizobia are host generalists. However, as stated, the objective of the present study was not to quantify host specificity or test for phylosymbiosis, but to evaluate the concordance of some metadata fields accompanying GenBank records. Host plant was therefore used as a standardized descriptor of isolation context rather than as a comprehensive measure of ecological similarity.

It is also unclear if geography is a meaningful indicator – no stats are done on geographic distances, country names only are reported in a qualitative way.

ANSWER: We thank the reviewer for raising this point. Our dataset contains country names as reported in GenBank metadata, but not precise geographic coordinates, which are not featured by the record fields. For these reasons, we treat “country” as a coarse descriptor of sampling origin rather than as a quantitative ecological variable. We agree that country identity alone cannot capture geographic distance or environmental similarity. However, in light of this comment, we performed a statistical check to evaluate whether identical 16S sequences tend to cluster geographically more than expected by chance. Using the observed country frequencies in our dataset (Fig. S3), we computed the expected probability that two randomly drawn records could originate from the same country. This probability resulted 0.12, whereas the observed proportion of identical-sequence pairs from the same country is 0.1077 (5,608 of 52,048). The observed value is extremely close to the expectation under random sampling, indicating that geographic origin does not appreciably structure 16S identity in this dataset. This supports our interpretation that “country” is best treated as metadata rather than as a proxy for ecological similarity. We have clarified this point in the revised manuscript.

A further consideration is that the following countries are political units of extremely heterogeneous size (e.g., China ~9.6 million km² vs. East Timor ~15,000 km²). Because countries differ by more than 600-fold in surface area and encompass highly diverse climates and ecosystems, “country” cannot be treated as a meaningful or comparable ecological or geographic unit. For this reason, we use country identity strictly as a coarse descriptor of sampling origin rather than as a proxy for geographic distance or environmental similarity.

About the above-discussed two aspects about host and country, summarizing the explanations, we agree with the Reviewer that host species identity and country of isolation should not be interpreted as direct or exhaustive proxies for microbial ecological similarity or biogeographic distance. In the revised manuscript, we therefore reframed these variables more cautiously as operational metadata descriptors available in GenBank, rather than as formal tests of phylosymbiosis or fine-scale geographic structure. Our aim was not to claim that identical 16S sequences must imply host specialization or local biogeographic clustering. Rather, we asked a more limited and technical question: whether identical sequences are associated with the same recorded host or geographic metadata more often than expected by chance within the database framework. In this sense, host and country were used as record-level contextual variables, not as claims about ecological equivalence.

To make this distinction clearer, we now emphasize that the observed concordance patterns describe how often identical 16S records retain the same host or country annotation in GenBank, and not whether those metadata are sufficient to define ecological similarity in a broader biological sense. This framing better reflects the scope of the study and the limitations of the available database metadata.

In addition to lack of quantitative analyses, the authors also lack clear descriptions of the current methods that were done. For example, the authors begin with finding all relevant 16S rRNA gene sequences. The exclude whole genome sequences. Why? These whole genomes could be mined for full length 16S sequences could they not?

ANSWER: We thank the Reviewer for this comment and for highlighting the need to clarify the methodological scope. As noted in the M&M section, the exclusion of whole-genome entries was deliberate, because the study was designed to evaluate the standard 16S-BLAST workflow on curated 16S records, rather than to reconstruct 16S loci from genome assemblies. In this sense, genome-based mining would address a different analytical question from the one posed here. Moreover, a 16S sequence recovered from a genome assembly would not necessarily share the same accession as the genome itself, since genome assemblies and 16S records are distinct NCBI entities.

As noted to Referee 2, we also briefly cross-referenced our NCBI-derived accession pool against GTDB as an external genome-based check for taxon attribution, which returned only 88 RefSeq matches. This limited overlap shows that genome-level coverage for the records under study is partial, and we used SILVA as more appropriate curated benchmark for species-level attribution in this 16S-based analysis.

In addition we can also clarify that the choice was based on two further and linked aspects: 1) The cases of whole genome records were a small minority over the individual sequences satisfying the reads length requirement; 2) The download and handling of a few megabase-long FASTA files to be matched with sequences spanning around 15 hundred kb would have been a laborious task with minimal margin of gain. In addition, since the focus here was on metadata information, we can also add that the comparison would have also had a possible bias because isolates which were deemed worthy of full genome sequencing by their depositing authors, especially in the past, tended to have a much more accurate characterization and identification when compared to loose single sequences from plain metabarcoding or Sanger approaches, that represent the overwhelming majority of the data hereby analyzed.

How do the authors know their second blast search strategy isn’t querying the same 16S sequences downloaded in step one?

ANSWER: Thank you for signaling to mention how this detail was dealt with. The second BLAST step was performed against the full NCBI nucleotide database, so it did not simply re-query the initial retrieval set. The ‘self matches’ were recognized and excluded from the counts by the presence of the identical accession number. This detail has been added

The authors also make several claims which are clear overstatements. For example - How does one know that an organism appearing more in BLAST is a more "globally successful organism" (Line 196)? Database bias should be acknowledged and carried as a major theme throughout this paper .

ANSWER: We thank the Reviewer for highlighting this point. We have removed the term “globally successful” and now describe these sequences as highly recurrent in the database. This wording better reflects the scope of our analysis and avoids misinterpreting database output as ecological success. Likewise, we agree on the need to tone down the meaning and extent of importance of the index and we have revised the sentences accordingly throughout the paper. We have better clarified, and added to the text, the fact that in essence, 1) in light of the human-applied labels of taxonomical attribution, that characterize the database records, this index is of pure physical nature (occurrence of DNA bases), being totally independent of the possible nomenclatural inconsistencies; (2) its significance as a taxon’s ecological prevalence and achieved genetic diversity, is at the same time linked also to the amount of scientific investigation effort devoted to the organisms and habitats which can feature the occurrence of that DNA sequence.

About database biases as addressed above in reply to a prior query, from Reviewer 2, we agree on the fact that, independently of our present purpose, different aspects need to be considered to properly frame the limits that public repositories pose to all studies that mine their content. This concept has been further remarked in the text and, in particular, by recapitulating their different structural limitations as evidenced by Keck, et al (2023). *Navigating the seven challenges of taxonomic reference databases in metabarcoding analyses.* Mol. Ecol. Res., 23, 742–755.

The authors also do not provide any of the scripts used for this paper. All R scripts should be made available on GitHub or similar platform for inspection. What packages were used? What version? What blast settings were used? All settings need to be documented so that another person could recreate the findings here.

ANSWER: We thank the Reviewer for raising this point. The analysis is not based on a hidden or undocumented workflow; rather, it is built from a sequence of standard, transparent steps implemented in separate files to manage the data volume. We have now made these materials explicit by providing the query-splitting R script, the BLAST batch file, and a workflow schematic, together with the software versions and command-line parameters needed for reproduction. This should make clear that the pipeline is straightforward to replicate once the documented inputs are followed.

The corresponding materials have been added to the revised Methods and as Supporting Information file S1Computational workflow and Reproducibility notes.

The authors also develop and show an h-index, but do not demonstrate how it is useful. For publication, one would need to add this demo.

ANSWER: We have added text to address such a point in order for any interested author to test the index for a taxon of choice. The potential usefulness of such measurement is to appreciate the level of dynamic sequence differentiation, replication spread, and persistence in the environment for a given organism at a given taxonomical rank level. Since the occurrence and availability of records in the public databases are proportional to the scientific focus and interest that each taxon has received from our side, the index is not suited for straight comparisons among taxa, as it would be affected by the study effort bias. The correction that can be recommended to enable a more direct evaluation between taxa is to normalize it by taking into account the number of database records used to calculate it.

In addition to this, there are several other concerns about the quality of the paper overall. First, citations seem to be mismatched to text.

ANSWER: The two websites referrals that were inserted in the text without a corresponding numbered presence in the list have been fixed. For all references, the matching with the cited article has been verified, and when necessary, the particular aspect that was quoted from a given paper has been further clarified in the text.

Second, the professionalism of some of the figures and text needs to be improved.

ANSWER: Fig. 2 has been redrawn to achieve a more professional style. The look of Fig. 3 (a screenshot of an excel spreadsheet), had been deemed functional to pointing out the calculation of the new concept and the fact that it refers to a bacterium, whose DNA sequence quantitative occurrence is *metaphorically* compared to an authors’ citation. If the Editor feels that it requires a restyling, we are ready to work on it.

Last, the AI use statement needs to be expanded to include version and usage in more detail.

ANSWER: We have expanded the AI use statement in the Methods to specify the provider, model version, and the exact scope of AI assistance (language editing only, no data analysis or generation of scientific claims), in line with PLOS ONE’s guidelines on AI transparency. The new version is the following:

“A generative AI assistant (Perplexity AI, version Pro featuring the integrated GPT‑5.1 OpenAI module, San Francisco, CA, USA) was employed to support the editorial preparation of the manuscript. Its use was restricted to language-related tasks (grammar correction, rephrasing for clarity, and streamlining of existing text), and no sections containing data analysis, results, or scientific interpretation were originally written by the tool. All outputs were manually checked, edited, and verified by the authors, who take full responsibility for the content. The use of AI tools does not affect the reproducibility of the study, which relies solely on the described computational pipeline and publicly available data..”

Are the claims properly placed in the context of the previous literature? Have the authors treated the literature fairly?
Throughout the introduction, the authors conflate the idea of three related, but distinct ideas (1) the use of 16S for use as a phylogenetic marker of vertical inheritance, (2) the assignment of taxonomic labels to the 16S gene sequence space, and (3) the mapping of the known ecology of the organism onto 16S state space. I believe the introduction warrants a major reframing.

ANSWER: We agree on the comment, and we see that the confounding aspect has been the order by which the topics had been put forward. We have now reorganized the section as outlined below.

In regards to (1): The 16S gene has a >50 year history to elucidate phylogenetic relationships. In the introduction, they should site the historic literature showing how this gene has been used for culture independent sequencing for over the last 50+ years, and authors should point to initial work.

ANSWER: We have accordingly opened the new introduction by citing the pioneering PNAS article from Carl Woese published in 1977 and continuing with two major contributions by Erko Stackebrandt (these citations are now listed as 14,15,16).

The authors also need to point to the large amount of work that has stated that the 16S gene is not accurate to establish species level relationships especially for short-read data.

ANSWER: Thanking the reviewer for this adequate recommendation, we have moved to this part of the introduction the Johnson et al. (2019) article appeared in Nature Communications 10(1), 5029, and has been cited, to date by 2716 articles, which explicitly proves that targeting individual hypervariable regions of the 16S gene using short-read sequencing platforms (like Illumina MiSeq) fundamentally lacks the taxonomic resolution required to achieve reliable species- or strain-level classification, which often requires full-length (~1500 bp) sequencing.

In addition, we cited the major review Clarridge (2004) Clinical Microbiology Reviews, 17(4), 840-862. A foundational article showing that while 16S rRNA is excellent for genus-level assignments, it lacks a consensus quantitative definition for defining a species due to low interspecies variability in certain genera. (Cited to date by 3475)

They cite the classic operational taxonomic unit (OTU) assignment of a 16S sequence to be considered a “different species” is 97%– but this is now a quite outdated idea with the advent of amplicon sequence variant (ASV) methods. These allow for one to determine ASV sequences to 100% of the sequence while controlling for errors (e.g. dada2). The authors use this outdated idea of OTUs to motivate their study objectives

ANSWER: This point has been perhaps misread. The only line in which we use the word OTU in the manuscript’s text is the sentence :*“ …reference databases, whose recommended format of analysis has shifted in time from the operational taxonomic units or OTUs to the amplicon sequence variants or ASVs [19,20].”* which is indeed in agreement with what the reviewer is reminding. Moreover, reference [20], published in this journal, is our own prior work (Fasolo A, Deb S, Stevanato P, Concheri G, Squartini A. *ASV vs OTUs clustering: Effects on alpha, beta, and gamma diversities in microbiome metabarcoding studies.* PLoS ONE. 2024;19(10):e0309065. <https://doi.org/10.1371/journal.pone.0309065>), which underlines our comprehension and contribution to this aspect.

The mention of 97 % in our manuscript, if that has been a confounding source, is instead appearing in the title of the cited reference from Robert Edgar (Edgar RC. *Updating the 97% identity threshold for 16S ribosomal RNA OTUs.* Bioinformatics. 2018;34(14):2371-2375.)

“When in such a process the BLAST algorithm yields sequences which are 100% identical to the searched query, one can suppose that, besides belonging supposedly to the same species, the organism being investigated and that of its matching subject record sequence should share a closer kinship between each other than with ones that have e.g. just 97.6 %, i.e. a situation where they are assigned to the same species but appear to represent two very diverged strains.”
In regards to (2). Taxonomic label assignment began with invention of the light microscope and the Linnaean taxonomic system and was based off of the morphology of organisms at first. One can imagine this was quite non-specific given that many unrelated microbes look similar under the light microscope! These have been updated extensively over history as tools develop - and most recently with genetic information derived from culture-independent sequencing, but taxonomy often times lags behind sequence information having historic misidentifications present. The authors mention that there were historic reclassification in taxonomic names which might be relevant to their analysis (line 268), but they do not correct for historic changes in taxonomic labels - if they exist.

ANSWER: We appreciate the relevance of the comment, and we thank the reviewer for the chance to explain the point and dispel the risk. The reclassifications that we cited in line 268 represent cases in which, beyond 16S -based attribution, a subsequent investigation involving either whole-genome or several loci for average nucleotide identity led to questioning the inclusion of an organism in its hitherto assumed taxonomic clade and warranted its transfer to another one and a name emendation. This however can affect one or more strains of a certain species but not necessarily the whole taxon. On the contrary, the simple change of taxonomic name as a consequence of refinements in the ecological /biochemical /physiological knowledge of that taxon is simply an update of its, often historical, original basonym, and in those cases, all isolates previously denominated under the prior name are migrated under the new name. Luckily, this is a service that has been regularly taken care of by the NCBI curators. As examples, all records that had been deposited as *Rhizobium meliloti* now bear the name *Sinorhizobium meliloti* in the organism field of the records (irrespective of the maintained original record title and of the possible papers cited in the other record fields). The same occurs for *Rhizobium hedysari*, which became *Rhizibium sullae*, etc. Therefore, we did not have biases to correct for what concerns changes of names from the original ones. While for the ‘horizontal’ changes across lineages due to reclassification there is no way to generalize corrections since that change could apply to sets of strains that embody a different subgroup to be split from the main group, which retains its identity.

In any event, the leading handle tool in our analysis was the DNA sequence itself, by which we sought the 100%-matching sequence mates, in a way that was therefore independent of nomenclatural inconsistencies.

In regards to (3). The authors interpret ecological dissimilarity between two closely related strains as a limitation to the 16S rDNA gene. However this framing applies the wrong tool to a well-understood phenomenon. The 16S gene is a slow molecular clock, ideal for understanding ancient relatedness between organisms, not fine scale ecological or functional differentiation. In contrast, many traits that determine ecological fitness in a particular environment are horizontally acquired genes (e.g. antibiotic resistance). Thus, the observed mismatch between ecology of the organism within the same taxonomic annotation may reflect two different processes happening within one genome.

ANSWER: We totally agree, but this is exactly what we are ourselves pointing out in the final part of the discussion on those results, whose text we recall here:

“*A metabarcoding sequence matching a database reference at 100% identity might derive from an organism with substantially different ecological properties [56,57]. Recommendations have been issued on the necessity that claims of species-level identification from short metabarcoding reads should be scrutinized [58]. All these patterns reflect the extraordinarily slow evolutionary rate of ribosomal RNA genes relative to ecologically significant traits encoded on mobile genetic elements. The mosaic genome architecture of rhizobia, with critical symbiotic determinants on transmissible plasmids subject to horizontal transfer, exemplifies how phylogeny and ecology can become decoupled. The higher importance of horizontal vs. vertical phylogeny in rhizobia and the risks of evolutionary interpretation biases when relying solely on the latter had been clearly pointed out in a key report already decades ago [59].In essence, this comprehensive mining of Rhizobium 16S rRNA sequences from GenBank has revealed profound disconnects between perfect sequence identity at this phylogenetic marker and concordance in taxonomic nomenclature, host plant associations, and geographic origins. The finding that nearly two-thirds of sequence pairs sharing 100% 16S identity exhibit complete discordance across all metadata categories provides an indirect numerical quantification of the risk of biases associated particularly as regards drawing ecological inferences from the straight use of 16S microbiome metabarcoding.* “

Therefore, in full alignment with the reviewer observation, we confirm that here we did not aim at proving that the same sequence should bear the same name and host and location in the NCBI records, but we asked this question (already assuming that it could turn out to be rhetorical for this system) precisely to point out that the database metadata would not actually support this assumption.

The authors should situate their idea in context of pangenomes – perhaps mentioning core, accessory, and shell genes and how this relates to their findings.

ANSWER: Regarding genome-wide approaches, as replied to a query from Reviewer 2, although full genome-based analyses such as ANI or whole-genome phylogeny fall outside the primary scope of the present study, which focuses on the practical use of 16S-based NCBI BLAST annotation, we added an external taxonomic comparison against the SILVA curated 16S rRNA framework (release 138.2). This comparison was not intended as a complete reclassification of the NCBI dataset, but rather as an external curated reference point to place the NCBI-based results in a broader taxonomic context and to assess whether stricter filtering improved taxonomic coherence. The corresponding text has been added in the Materials and Methods section, the Results section in the paragraph following Tables 4 and 5, and the Discussion section where the practical use and limits of 16S-based annotation are discussed.

We also cross-referenced our NCBI-derived accession pool against GTDB as an external genome-based check. This search returned only 88 RefSeq matches, indicating that genome-level coverage for the records under study is limited. Because our question concerns the taxonomic interpretation of 16S records rather than genome mining per se, we only complemented our check with SILVA for resolving species-level attribution.

In conclusion, we are aligned with the Reviewers’ observation that, differently from metadata label commonalities, comparisons from the full genomic information would provide grounds for solid ecological similarity and a phylogenetical relatedness.

In summary, the paper needs to:
-Consider that short-read 16S cannot distinguish reliably to the species level with high confidence, and this is well known.

ANSWER: Again, this was also our point. We have better clarified it thanks to the reviewer’s suggestion to include new references [17.18] stressing the limited discriminatory power of short reads. This concept was in the first place the reason to set a minimum sequence length range for the data mining and usable records retrieval (“*The sequence length constraint of 1200 to 1600 base pairs was implemented to exclude partial sequences that might yield unreliable BLAST results while accommodating natural variation in 16S gene length among different bacterial lineages [32]*”)

-Consider - or at least comment on - if correction for historical changes in taxonomic reclassifications were done during analyses.

ANSWER: as anticipated in a reply to a query above, this aspect did not require a correction as the update of nomenclature in the organism field of the records belongs to the customary actions that NCBI carries out whenever the news are published in the official taxonomy reports.

Anyhow, our overall policy in this demonstrative survey was to use data exactly as they are. We wanted to highlight that the data and their respective metadata appearing in the sequence bank are not reliable for ecological meta-analytic interpretations. As a secondary line of findings, we then could add the suggestion that the microbial h-index instead could evaluate the success of a sequence independently of its labels of name or other aspects.

-Consider that it might not be a reasonable proxy to use plant host species as the best marker of microbial species trait similarity.

ANSWER: We are once again on the same line of thoughts; the plant category was used precisely to highlight its potential discrepancies.

-Consider that geographic country of origin might not be the best marker of microbial species ecologic similarity. Plants are transported and distributed globally.

ANSWER: Idem as above for plants and names; we used the category to challenge and probe the potential discordances. As regards this specific observation, however, a distinction is needed. While plants, as a single biont, could potentially display a certain degree of cosmopolitism, when it comes to the interaction between macro- and microsymbiont pairs, the constraint of host range exerts a major selective gate effect and sharply restricts the possible combinations.

-Develop a statistical framework to answer their question quantitatively, not just qualitatively.

ANSWER: As outlined also in reply to Reviewer. 2, we have thoroughly implemented the analysis with an independent statistical backup, (see new Fig.1 and three new tables (Table 4 Table 5 and Table S1). For this purpose, we strengthened the analytical framework by introducing a non-parametric null model based on 10,000 Monte Carlo permutations without replacement, designed to preserve the marginal frequencies of taxon, host, and country metadata while randomizing only the query-subject pairing. This framework was applied to both the broad metadata-complete dataset (dataset B; 52,048 matches) and to a more stringently curated subset (dataset C; 11,422 matches), allowing us to test whether the observed concordance patterns differed from random expectation. These additions were incorporated in the Materials and Methods section under “Metadata extraction, cleaning and standardization” and the corresponding results are now reported in the Results section in the paragraphs introducing Tables 4 and 5, as well as in the opening part of the Discussion.

-Demonstrate clearly the novel insight the h-index gives.

ANSWER: As commented in reply to a comment from Reviewer 2, agreeing on the recommendation to avoid overinterpreting its biological significance and presenting it more cautiously as a preliminary exploratory concept, we can say the following. We are aligned on this caveat, and we have accordingly re-toned the definition and stated its boundaries of usefulness. In the text we have better clarified that its significance as a taxon’s ecological prevalence and achieved genetic diversity is linked also to the amount of scientific investigation effort devoted to the organisms and habitats which can feature the occurrence of that DNA sequence. Nonetheless, at the same time, despite the human-applied labels of taxonomical attribution that characterize the database records, this index of pure physical nature (occurrence of DNA bases), is totally independent of any of the possible nomenclatural inconsistencies.

Hoping to have fulfilled all the issues, we thank the Reviewers and Editor for their constructive criticism that led to a substantial improvement of the present report.

Looking forward to hearing from you, thank you for your attention.

On behalf of all coauthors

Andrea Squartini
