# Supplementary material for: GenBank mining reveals novel insights into Rhizobium phylogeny: Identical 16S rRNA sequences are mainly uncoupled from species designation, host plant, and geographic origin: How this search suggested the definition of a direct ‘microbial h-index’
Source: PLoS One. 2026 Sep 11;21(9):e0357973. doi: 10.1371/journal.pone.0357973 (PMC13567781; doi:10.1371/journal.pone.0357973)
Supplement: Supplementary file 4 [file pone.0357973.s005.docx]

Additional Editor Comments :

Dear Andrea,

The revised manuscript has substantially improved, and the main concerns raised during the previous review have been addressed. Only a few minor revisions are required before acceptance.

Please replace expressions such as “ecological success”, “fitness”, “productivity”, and “pangenomic diversity” with more cautious wording, for example: “a database-level metric of sequence recurrence and representation.”

These terms currently imply a biological meaning that is not directly demonstrated by sequence recurrence in GenBank and may also be affected by sampling and research effort.

ANSWER: We agree, and we have changed the terminology as follows:

In substitution of the expression “ecological success” we have used the following: “a database-level metric of sequence recurrence and representation”; “recurrence proficiency”; “a given taxonomically-linked sequence representation”; “individual duplication and persistence proficiency”

In substitution of the word “fitness”: we have used the following: “individual genotype growth”; “adaptability”.

In substitution of the word “productivity”: we have used the term “reproductive potentiality”

The expression “pangenomic diversity” has been removed.

- Abstract

Please replace:

“potential ecological prevalence, pangenomic diversity and spread”**

with, for example: “sequence recurrence, representation and distribution within public databases.”

In particular, “pangenomic diversity” is not directly assessed in the present study.

ANSWER: The suggested substitution has been made.

- Conclusions on 16S rRNA

Please replace:

**“increasingly weakening reliability of 16S-based species identification”**

with a more specific formulation such as:

**“limitations of relying on 16S rRNA alone for fine-scale taxonomic and metadata-based inference.”**

The present results support this more specific conclusion rather than a general statement on the overall reliability of 16S rRNA.

ANSWER: The suggested substitution has been made.

- Clonality terminology

Please replace “clonal kins” and “clonal relationships” with:

“sequences sharing 100% 16S identity”

or

“identical 16S sequences.”

Identical 16S sequences do not necessarily imply genome-wide clonality.

ANSWER: The words “clonal” and “kins” have been removed

- Metadata standardization

Please replace:

“to eliminate taxonomic, botanical, and geographic assignment errors”

with:

“to reduce nomenclatural, formatting, and annotation inconsistencies.”

The standardization procedure can reduce inconsistencies in database records but cannot establish that the original biological assignments are correct.

ANSWER: The suggested substitution has been made.

Reviewers' comments:

Reviewer's Responses to Questions

Comments to the Author

1. If the authors have adequately addressed your comments raised in a previous round of review and you feel that this manuscript is now acceptable for publication, you may indicate that here to bypass the “Comments to the Author” section, enter your conflict of interest statement in the “Confidential to Editor” section, and submit your "Accept" recommendation.

Reviewer #1: All comments have been addressed

Reviewer #2: All comments have been addressed

________________________________________

2. Is the manuscript technically sound, and do the data support the conclusions?

The manuscript must describe a technically sound piece of scientific research with data that supports the conclusions. Experiments must have been conducted rigorously, with appropriate controls, replication, and sample sizes. The conclusions must be drawn appropriately based on the data presented.

Reviewer #1: Yes

Reviewer #2: Yes

________________________________________

3. Has the statistical analysis been performed appropriately and rigorously?

Reviewer #1: Yes

Reviewer #2: Yes

________________________________________

4. Have the authors made all data underlying the findings in their manuscript fully available?

The PLOS Data policy requires authors to make all data underlying the findings described in their manuscript fully available without restriction, with rare exception (please refer to the Data Availability Statement in the manuscript PDF file). The data should be provided as part of the manuscript or its supporting information, or deposited to a public repository. For example, in addition to summary statistics, the data points behind means, medians and variance measures should be available. If there are restrictions on publicly sharing data—e.g. participant privacy or use of data from a third party—those must be specified.

Reviewer #1: Yes

Reviewer #2: Yes

________________________________________

5. Is the manuscript presented in an intelligible fashion and written in standard English?

PLOS ONE does not copyedit accepted manuscripts, so the language in submitted articles must be clear, correct, and unambiguous. Any typographical or grammatical errors should be corrected at revision, so please note any specific errors here.

Reviewer #1: Yes

Reviewer #2: Yes

________________________________________

6. Review Comments to the Author

Please use the space provided to explain your answers to the questions above. You may also include additional comments for the author, including concerns about dual publication, research ethics, or publication ethics. (Please upload your review as an attachment if it exceeds 20,000 characters)

Reviewer #1: The authors have satisfactorily addressed all of the reviewers’ comments. The revisions have significantly improved the scientific rigor, clarity, and overall quality of the manuscript. In my opinion, the manuscript is now suitable for publication and can be accepted in its current form.

Reviewer #2: The authors have adequately addressed the concerns raised during the previous round of review. The manuscript has been clearly improved, particularly with the addition of statistical analyses, better clarification of the methodology, and a more balanced interpretation of the findings and study limitations.

The authors have also provided detailed responses to the reviewers’ comments and made appropriate changes to the manuscript. I believe the revised version is scientifically stronger and the remaining limitations do not affect the main conclusions of the study.

The manuscript is suitable for publication in its current form.

Hoping to have addressed all the pending issues, we thank you for your kind attention, and we look forward to hearing from you.
