# Supplementary material for: GenBank mining reveals novel insights into Rhizobium phylogeny: Identical 16S rRNA sequences are mainly uncoupled from species designation, host plant, and geographic origin: How this search suggested the definition of a direct ‘microbial h-index’
Source: PLoS One. 2026 Sep 11;21(9):e0357973. doi: 10.1371/journal.pone.0357973 (PMC13567781; doi:10.1371/journal.pone.0357973)
Supplement: Peer review history

# Peer review: Decision Letter 0

**DOI:** 10.1371/journal.pone.0357973.r001

**Authors:** Luigimaria Borruso

29 Apr 2026

Dear Dr. Squartini,

Thank you for submitting your manuscript to PLOS ONE. After careful consideration, we feel that it has merit but does not fully meet PLOS ONE’s publication criteria as it currently stands. Therefore, we invite you to submit a revised version of the manuscript that addresses the points raised during the review process.

Please submit your revised manuscript by Jun 13 2026 11:59PM. If you will need more time than this to complete your revisions, please reply to this message or contact the journal office at plosone@plos.org. When you're ready to submit your revision, log on to https://www.editorialmanager.com/pone/ and select the 'Submissions Needing Revision' folder to locate your manuscript file.

- A letter that responds to each point raised by the academic editor and reviewer(s). You should upload this letter as a separate file labeled 'Response to Reviewers'.
- A marked-up copy of your manuscript that highlights changes made to the original version. You should upload this as a separate file labeled 'Revised Manuscript with Track Changes'.
- An unmarked version of your revised paper without tracked changes. You should upload this as a separate file labeled 'Manuscript'.

If you would like to make changes to your financial disclosure, please include your updated statement in your cover letter. Guidelines for resubmitting your figure files are available below the reviewer comments at the end of this letter.

If applicable, we recommend that you deposit your laboratory protocols in protocols.io to enhance the reproducibility of your results. Protocols.io assigns your protocol its own identifier (DOI) so that it can be cited independently in the future. For instructions see: https://journals.plos.org/plosone/s/submission-guidelines#loc-laboratory-protocols. Additionally, PLOS ONE offers an option for publishing peer-reviewed Lab Protocol articles, which describe protocols hosted on protocols.io. Read more information on sharing protocols at https://plos.org/protocols?utm_medium=editorial-email&utm_source=authorletters&utm_campaign=protocols.

As the corresponding author, your ORCID iD is verified in the submission system and will appear in the published article. PLOS supports the use of ORCID, and we encourage all coauthors to register for an ORCID iD and use it as well. Please encourage your coauthors to verify their ORCID iD within the submission system before final acceptance, as unverified ORCID iDs will not appear in the published article. *Only*  the individual author can complete the verification step; PLOS staff *cannot*  verify ORCID iDs on behalf of authors.

We look forward to receiving your revised manuscript.

Kind regards,

Luigimaria Borruso

Academic Editor

PLOS One

**Journal Requirements:**

1. When submitting your revision, we need you to address these additional requirements.

Please ensure that your manuscript meets PLOS ONE's style requirements, including those for file naming. The PLOS ONE style templates can be found at

https://journals.plos.org/plosone/s/file?id=wjVg/PLOSOne_formatting_sample_main_body.pdf and

https://journals.plos.org/plosone/s/file?id=ba62/PLOSOne_formatting_sample_title_authors_affiliations.pdf

2. Please note that PLOS One has specific guidelines on code sharing for submissions in which author-generated code underpins the findings in the manuscript. In these cases, we expect all author-generated code to be made available without restrictions upon publication of the work. Please review our guidelines at https://journals.plos.org/plosone/s/materials-and-software-sharing#loc-sharing-code and ensure that your code is shared in a way that follows best practice and facilitates reproducibility and reuse.

3. Thank you for stating the following financial disclosure:

This wark was made possible in part by funding from University of Padova, Grant DOR-DAFNAE 2023 to Andrea Squartini.

Please state what role the funders took in the study.  If the funders had no role, please state: "The funders had no role in study design, data collection and analysis, decision to publish, or preparation of the manuscript."

If this statement is not correct you must amend it as needed.

Please include this amended Role of Funder statement in your cover letter; we will change the online submission form on your behalf.

4. Please upload a new copy of Figure 3, as the detail is not clear. Please follow the link for more information:  https://journals.plos.org/plosone/s/figures

5. We note that there is identifying data in the Supporting Information file <Supporting Information file S1>. Due to the inclusion of these potentially identifying data, we have removed this file from your file inventory. Prior to sharing human research participant data, authors should consult with an ethics committee to ensure data are shared in accordance with participant consent and all applicable local laws.

Data sharing should never compromise participant privacy. It is therefore not appropriate to publicly share personally identifiable data on human research participants. The following are examples of data that should not be shared:

-Name, initials, physical address

-Ages more specific than whole numbers

-Internet protocol (IP) address

-Specific dates (birth dates, death dates, examination dates, etc.)

-Contact information such as phone number or email address

-Location data

-ID numbers that seem specific (long numbers, include initials, titled “Hospital ID”) rather than random (small numbers in numerical order)

Data that are not directly identifying may also be inappropriate to share, as in combination they can become identifying. For example, data collected from a small group of participants, vulnerable populations, or private groups should not be shared if they involve indirect identifiers (such as sex, ethnicity, location, etc.) that may risk the identification of study participants.

Additional guidance on preparing raw data for publication can be found in our Data Policy (https://journals.plos.org/plosone/s/data-availability#loc-human-research-participant-data-and-other-sensitive-data) and in the following article: http://www.bmj.com/content/340/bmj.c181.long.

Please remove or anonymize all personal information (<specific identifying information in file to be removed>), ensure that the data shared are in accordance with participant consent, and re-upload a fully anonymized data set. Please note that spreadsheet columns with personal information must be removed and not hidden as all hidden columns will appear in the published file.

6. If the reviewer comments include a recommendation to cite specific previously published works, please review and evaluate these publications to determine whether they are relevant and should be cited. There is no requirement to cite these works unless the editor has indicated otherwise. 

[Note: HTML markup is below. Please do not edit.]

Reviewers' comments:

**Comments to the Author**

1. Is the manuscript technically sound, and do the data support the conclusions?

Reviewer #1: Yes

Reviewer #2: Yes

Reviewer #3: No

**********

2. Has the statistical analysis been performed appropriately and rigorously?

Reviewer #1: N/A

Reviewer #2: I Don't Know

Reviewer #3: No

**********

3. Have the authors made all data underlying the findings in their manuscript fully available?

The PLOS Data policy

Reviewer #1: Yes

Reviewer #2: Yes

Reviewer #3: No

**********

4. Is the manuscript presented in an intelligible fashion and written in standard English?

Reviewer #1: Yes

Reviewer #2: Yes

Reviewer #3: Yes

**********

**Reviewer #1:**  The findings of this study critically highlight the increasing call for taxonomic approaches that go beyond the 16S rRNA gene, integrating phenotypic and ecological data. The manuscript also introduces a particularly innovative approach by applying the h-index concept to “citations” of sequences, providing a useful metric to assess the ecological prevalence and global distribution of bacterial taxa.

The manuscript is well written, clear, and logically organized, with an English level in line with the journal’s standards.

The "Materials and Methods" section is detailed and easy to follow. As a minor recommendation, I suggest including a diagram or figure to illustrate the analytical workflow to improve the clarity for the readers.

The Results are clearly presented, and the interpretations in the Discussion are consistent logic. The Conclusions are well supported by the data.

Overall, given its novelty, I suggest accepting this manuscript for publication.

**Reviewer #2:**  Greetings

Very good work. But kindly edit the points below:

Major Comments

1. Lack of Robust Statistical Analysis

The study relies primarily on descriptive statistics (percentages and counts), without applying inferential statistical tests to support the conclusions.

For example, the reported high proportion of discordance among identical sequences is intriguing, but it is unclear whether this observation significantly deviates from what would be expected by chance.

The authors should:

Apply appropriate statistical tests (e.g., chi-square tests, permutation tests, or null model comparisons).

Provide confidence intervals where applicable.

Consider modeling approaches to quantify associations between sequence identity and metadata concordance.

Without this, the conclusions remain observational rather than analytically supported.

2. Reliability and Bias of GenBank Metadata

The study heavily depends on metadata extracted from GenBank records (species name, host, country), yet these fields are known to be inconsistent, incomplete, and sometimes inaccurate.

Although the authors filtered the dataset to include only entries with complete metadata, there is no critical discussion of:

Potential annotation errors

Inconsistent taxonomic assignments

Sampling and submission bias

This is a major limitation that could significantly influence the results.

The manuscript would benefit from:

A dedicated paragraph discussing metadata reliability

A sensitivity analysis or validation approach, if possible

3. Absence of Genomic-Level Validation

A central claim of the manuscript is that 16S rRNA identity is insufficient for reflecting ecological and taxonomic relationships. While this is a recognized issue, the study does not provide any comparison with genome-based approaches.

Including discussion or comparison with:

Average Nucleotide Identity (ANI)

Genome Taxonomy Database (GTDB)

Whole-genome phylogeny

would greatly strengthen the argument and place the findings in a broader modern taxonomic context.

4. Interpretation of Results is Sometimes Overstated

The manuscript occasionally presents conclusions in a way that may overgeneralize the findings.

While the data clearly highlight limitations of 16S-based inference at fine resolution, it is important to acknowledge that:

16S rRNA remains useful for higher-level classification (e.g., genus-level)

Its limitations are context-dependent

The conclusions should be revised to reflect a more balanced and nuanced interpretation.

5. The “Microbial h-index” Concept Requires Caution

The proposed “microbial h-index” is an interesting and creative idea. However, in its current form, it appears largely conceptual and lacks biological validation.

Before presenting it as a meaningful ecological or evolutionary metric, the authors should:

Clarify its limitations

Avoid overinterpreting its biological significance

Present it more cautiously as a preliminary or exploratory concept

Kind regards.

**Reviewer #3:** ========

What are the main claims of the paper and how significant are they for the discipline?

========

The authors main claims are to use the Rhizobium genus to examine concordance of microbial species taxonomies and ecological similarities with 16S rDNA sequence. They frame their results as a “challenge [to] the increasingly weakening reliability of 16S based species identification in capturing the true functional and ecological diversity of bacteria”. They also introduce a quantitative (h-index) described as an indicator of a taxon’s ecological prevalence and genomic diversity, which is independent of nomenclatural inconsistencies.

Their proposed scientific question at the end of the introduction is: “Under such premises, having chosen the Rhizobium genus as a suitable case to probe the system, the question we addressed was: should one expect as more likely that, when 16S sequences share 100% identity (‘clonal kins’) they: a) were recorded with the same taxonomic name; b) were isolated from closer rather than distant sites; c) were isolated from nodules of the same host plant ?”

Rhizobium is an important bacterial genus with implications for One Health, crop productivity, ecosystem nitrogen cycling, and sustainable agriculture, making this genus an intensively studied and important bacterial group for human enterprise. In addition, methods which utilize already existing data in publicly available databases are important contributions to the scientific community.

========

Do the data and analyses fully support the claims? If not, what other evidence is required?

========

The analyses do not support the author's claims or question. The paper is only qualitative in nature, and no statistical framework is employed to test the author's hypothesis. This leaves the main questions (line 124) “should one expect as more likely that, when 16S sequences share 100% identity they (a) were recorded with the same taxonomic name, (b) were isolated from closer rather than distant sites (c) were isolated from nodules of the same host plant?” unanswered. Because the authors constrain the analysis only to sequences with 100% identity, they do not have the appropriate dataset to support statistical analysis of their proposed framework. For publication, the authors would need to develop and employ such a statistical framework.

In general, throughout the paper, only descriptive qualitative analyses are reported. The authors seem to use subspecies mismatches as evidence of species-level discordance. This would need to be corrected.

The authors propose that a measure of ecological similarity between two microbes is the host species identity from which they are isolated, but it is unclear if this is an appropriate indicator of microbial ecological similarity for the system in question. Some species are known to be generalists and might exist in multiple hosts. No statistical analysis was done to look at host relatedness and microbial relatedness for evidence of phylosymbiosis.

It is also unclear if geography is a meaningful indicator – no stats are done on geographic distances, country names only are reported in a qualitative way.

In addition to lack of quantitative analyses, the authors also lack clear descriptions of the current methods that were done. For example, the authors begin with finding all relevant 16S rRNA gene sequences. The exclude whole genome sequences. Why? These whole genomes could be mined for full length 16S sequences could they not? How do the authors know their second blast search strategy isn’t querying the same 16S sequences downloaded in step one?

The authors also make several claims which are clear overstatements. For example - How does one know that an organism appearing more in BLAST is a more "globally successful organism" (Line 196)? Database bias should be acknowledged and carried as a major theme throughout this paper .

The authors also do not provide any of the scripts used for this paper. All R scripts should be made available on GitHub or similar platform for inspection. What packages were used? What version? What blast settings were used? All settings need to be documented so that another person could recreate the findings here.

The authors also develop and show an h-index, but do not demonstrate how it is useful. For publication, one would need to add this demo.

In addition to this, there are several other concerns about the quality of the paper overall. First citations seem to be mismatched to text. Second, the professionalism of some of the figures and text needs to be improved. Last, the AI use statement needs to be expanded to include version and usage in more detail.

Are the claims properly placed in the context of the previous literature? Have the authors treated the literature fairly?

Throughout the introduction, the authors conflate the idea of three related, but distinct ideas (1) the use of 16S for use as a phylogenetic marker of vertical inheritance, (2) the assignment of taxonomic labels to the 16S gene sequence space, and (3) the mapping of the known ecology of the organism onto 16S state space. I believe the introduction warrants a major reframing.

In regards to (1): The 16S gene has a >50 year history to elucidate phylogenetic relationships. In the introduction, they should site the historic literature showing how this gene has been used for culture independent sequencing for over the last 50+ years, and authors should point to initial work. The authors also need to point to the large amount of work that has stated that the 16S gene is not accurate to establish species level relationships especially for short-read data. They cite the classic operational taxonomic unit (OTU) assignment of a 16S sequence to be considered a “different species” is 97%– but this is now a quite outdated idea with the advent of amplicon sequence variant (ASV) methods. These allow for one to determine ASV sequences to 100% of the sequence while controlling for errors (e.g. dada2). The authors use this outdated idea of OTUs to motivate their study objectives

“When in such a process the BLAST algorithm yields sequences which are 100% identical to the searched query, one can suppose that, besides belonging supposedly to the same species, the organism being investigated and that of its matching subject record sequence should share a closer kinship between each other than with ones that have e.g. just 97.6 %, i.e. a situation where they are assigned to the same species but appear to represent two very diverged strains.”

In regards to (2). Taxonomic label assignment began with invention of the light microscope and the Linnaean taxonomic system and was based off of the morphology of organisms at first. One can imagine this was quite non-specific given that many unrelated microbes look similar under the light microscope! These have been updated extensively over history as tools develop - and most recently with genetic information derived from culture-independent sequencing, but taxonomy often times lags behind sequence information having historic misidentifications present. The authors mention that there were historic reclassification in taxonomic names which might be relevant to their analysis (line 268), but they do not correct for historic changes in taxonomic labels - if they exist.

In regards to (3). The authors interpret ecological dissimilarity between two closely related strains as a limitation to the 16S rDNA gene. However this framing applies the wrong tool to a well-understood phenomenon. The 16S gene is a slow molecular clock, ideal for understanding ancient relatedness between organisms, not fine scale ecological or functional differentiation. In contrast, many traits that determine ecological fitness in a particular environment are horizontally acquired genes (e.g. antibiotic resistance). Thus, the observed mismatch between ecology of the organism within the same taxonomic annotation may reflect two different processes happening within one genome. The authors should situate their idea in context of pangenomes – perhaps mentioning core, accessory, and shell genes and how this relates to their findings.

In summary, the paper needs to:

-Consider that short-read 16S cannot distinguish reliably to the species level with high confidence, and this is well known.

-Consider - or at least comment on - if correction for historical changes in taxonomic reclassifications were done during analyses.

-Consider that it might not be a reasonable proxy to use plant host species as the best marker of microbial species trait similarity.

-Consider that geographic country of origin might not be the best marker of microbial species ecologic similarity. Plants are transported and distributed globally.

-Develop a statistical framework to answer their question quantitatively, not just qualitatively.

-Demonstrate clearly the novel insight the h-index gives.

**********

what does this mean?). If published, this will include your full peer review and any attached files.

If you choose “no”, your identity will remain anonymous but your review may still be made public.

**Do you want your identity to be public for this peer review?**  For information about this choice, including consent withdrawal, please see our Privacy Policy

Reviewer #1: No

Reviewer #2: No

Reviewer #3: No

**********

[NOTE: If reviewer comments were submitted as an attachment file, they will be attached to this email and accessible via the submission site. Please log into your account, locate the manuscript record, and check for the action link "View Attachments". If this link does not appear, there are no attachment files.]

To ensure your figures meet our technical requirements, please review our figure guidelines: https://journals.plos.org/plosone/s/figures

You may also use PLOS’s free figure tool, NAAS, to help you prepare publication quality figures: https://journals.plos.org/plosone/s/figures#loc-tools-for-figure-preparation.

NAAS will assess whether your figures meet our technical requirements by comparing each figure against our figure specifications.

# Author comment: Author response to Decision Letter 1

**DOI:** 10.1371/journal.pone.0357973.r002

1 Jul 2026

Reviewer #1: The findings of this study critically highlight the increasing call for taxonomic approaches that go beyond the 16S rRNA gene, integrating phenotypic and ecological data. The manuscript also introduces a particularly innovative approach by applying the h-index concept to “citations” of sequences, providing a useful metric to assess the ecological prevalence and global distribution of bacterial taxa.

The manuscript is well written, clear, and logically organized, with an English level in line with the journal’s standards.

The "Materials and Methods" section is detailed and easy to follow. As a minor recommendation, I suggest including a diagram or figure to illustrate the analytical workflow to improve the clarity for the readers.

The Results are clearly presented, and the interpretations in the Discussion are consistent logic. The Conclusions are well supported by the data.

Overall, given its novelty, I suggest accepting this manuscript for publication.

ANSWER: We appreciate these positive remarks about quality and novelty, as well as for endorsing publication. As suggested, we have added a workflow diagram scheme, depicting all the analytical phases of the study, including sequence retrieval, iterative BLAST analysis, metadata extraction and cleaning, statistical concordance analysis, and calculation of diversity metrics. This graphical outline now also includes all the new analyses, which have been added for the verification of the statistical significance of the results by comparing those of a null model and further checks. Given the importance that reviewers have signalled for the inclusion of these control analyses, we propose that this outline could become a new figure (New Fig.1).

Reviewer #2: Greetings

Very good work. But kindly edit the points below:

Major Comments

1. Lack of Robust Statistical Analysis

The study relies primarily on descriptive statistics (percentages and counts), without applying inferential statistical tests to support the conclusions.

For example, the reported high proportion of discordance among identical sequences is intriguing, but it is unclear whether this observation significantly deviates from what would be expected by chance.

The authors should:

Apply appropriate statistical tests (e.g., chi-square tests, permutation tests, or null model comparisons).

Provide confidence intervals where applicable.

Consider modeling approaches to quantify associations between sequence identity and metadata concordance.

Without this, the conclusions remain observational rather than analytically supported.

ANSWER: First, we thank the reviewer for opening the report with the positive recognition Very good work. We equally acknowledge the relevant comments about implementing the analysis with an appropriate independent statistical backup, and, as in part anticipated above, for this purpose we strengthened the analytical framework by introducing a non-parametric null model based on 10,000 Monte Carlo permutations without replacement, designed to preserve the marginal frequencies of taxon, host, and country metadata while randomizing only the query-subject pairing. This framework was applied to both the broad metadata-complete dataset (dataset B; 52,048 matches) and to a more stringently curated subset (dataset C; 11,422 matches), allowing us to test whether the observed concordance patterns differed from random expectation. These additions were incorporated in the Materials and Methods section under “Metadata extraction, cleaning and standardization” and the corresponding results are now reported in the Results section in the paragraphs introducing Tables 4 and 5, as well as in the opening part of the Discussion.

The repetition of the analysis on updated data led to slight modifications in the numbers and proportions, leading also to a new revised version of the prior Fig. 2

2. Reliability and Bias of GenBank Metadata

The study heavily depends on metadata extracted from GenBank records (species name, host, country), yet these fields are known to be inconsistent, incomplete, and sometimes inaccurate.

Although the authors filtered the dataset to include only entries with complete metadata, there is no critical discussion of:

Potential annotation errors

Inconsistent taxonomic assignments

Sampling and submission bias

This is a major limitation that could significantly influence the results.

The manuscript would benefit from:

A dedicated paragraph discussing metadata reliability

A sensitivity analysis or validation approach, if possible

ANSWER: We agree with the Reviewer that GenBank metadata can contain inconsistencies, incomplete annotations, and historical nomenclatural ambiguity. To address this issue, we explicitly added a metadata-cleaning and standardization step in the revised Materials and Methods, including harmonization of organism names, host plant names, and country designations, and we introduced a more conservative high-resolution subset (dataset C) in which taxonomic assignments were explicitly resolved for both query and subject. In addition, the revised Discussion now contains a dedicated interpretation of how unresolved taxonomy contributes to apparent overlap in dataset B, whereas stricter curation in dataset C reduces annotation noise and reveals a clearer biological signal.

About the inherent weaknesses of the available databases, we agree on the fact that, independently of our present purpose, different aspects need to be considered to properly frame the limits that public repositories pose to all studies that mine their content. This concept has been further remarked in the text and, in particular, by recapitulating their different structural limitations as evidenced by Keck, et al (2023). Navigating the seven challenges of taxonomic reference databases in metabarcoding analyses. Mol. Ecol. Res., 23, 742–755.

3. Absence of Genomic-Level Validation

A central claim of the manuscript is that 16S rRNA identity is insufficient for reflecting ecological and taxonomic relationships. While this is a recognized issue, the study does not provide any comparison with genome-based approaches.

Including discussion or comparison with:

Average Nucleotide Identity (ANI)

Genome Taxonomy Database (GTDB)

Whole-genome phylogeny

would greatly strengthen the argument and place the findings in a broader modern taxonomic context.

ANSWER: We thank the Reviewer for this constructive suggestion. Although full genome-based analyses such as ANI or whole-genome phylogeny fall outside the primary scope of the present study, which focuses on the practical use of 16S-based NCBI BLAST annotation, we added an external taxonomic comparison against the SILVA curated 16S rRNA framework (release 138.2). This comparison was not intended as a complete reclassification of the NCBI dataset, but rather as an external curated reference point to place the NCBI-based results in a broader taxonomic context and to assess whether stricter filtering improved taxonomic coherence. The corresponding text has been added in the Materials and Methods section, the Results section in the paragraph following Tables 4 and 5, and the Discussion section where the practical use and limits of 16S-based annotation are discussed.

We also cross-referenced our NCBI-derived accession pool against GTDB as an external genome-based check. This search returned only 88 RefSeq matches, indicating that genome-level coverage for the records under study is limited. Because our question concerns the taxonomic interpretation of 16S records rather than genome mining per se, we only complemented our check with SILVA for resolving species-level attribution.

4. Interpretation of Results is Sometimes Overstated

The manuscript occasionally presents conclusions in a way that may overgeneralize the findings.

While the data clearly highlight limitations of 16S-based inference at fine resolution, it is important to acknowledge that:

16S rRNA remains useful for higher-level classification (e.g., genus-level)

Its limitations are context-dependent

The conclusions should be revised to reflect a more balanced and nuanced interpretation.

ANSWER: We appreciate this observation and have revised the Discussion to present a more balanced interpretation. In particular, we now explicitly state that, although 16S rRNA does not resolve fine-scale taxonomic or ecological differences, it remains a widely used marker for rapid database-based identification and continues to be broadly applicable to the many historical and environmental accessions that lack genome sequences. We also clarified that our results do not question the value of genome-based taxonomy but rather highlight the practical limits of relying on 16S alone when annotation quality, host information, and geographic metadata are taken into account.

5. The “Microbial h-index” Concept Requires Caution

The proposed “microbial h-index” is an interesting and creative idea. However, in its current form, it appears largely conceptual and lacks biological validation.

Before presenting it as a meaningful ecological or evolutionary metric, the authors should:

Clarify its limitations

Avoid overinterpreting its biological significance

Present it more cautiously as a preliminary or exploratory concept

ANSWER: We are aligned on this caveat, and we have accordingly re-toned the definition and stated its boundaries of usefulness. We have better clarified that, in essence; 1) in spite of the human-applied labels of taxonomical attribution, that characterize the database records, this index is of pure physical nature (occurrence of DNA bases), thus being totally independent of the possible nomenclatural inconsistencies; (2) its significance as a taxon’s ecological prevalence and achieved genetic diversity, is at the same time linked also to the amount of scientific investigation effort devoted to the organisms and habitats which can feature the occurrence of that DNA sequence. This aspect can call for a normalization (e.g., dividing by the number of database records of the taxon of choice), in case one would aim at comparing the indices of different organisms.

Reviewer #3: ========

What are the main claims of the paper and how significant are they for the discipline?

========

The authors main claims are to use the Rhizobium genus to examine concordance of microbial species taxonomies and ecological similarities with 16S rDNA sequence. They frame their results as a “challenge [to] the increasingly weakening reliability of 16S based species identification in capturing the true functional and ecological diversity of bacteria”. They also introduce a quantitative (h-index) described as an indicator of a taxon’s ecological prevalence and genomic diversity, which is independent of nomenclatural inconsistencies.

Their proposed scientific question at the end of the introduction is: “Under such premises, having chosen the Rhizobium genus as a suitable case to probe the system, the question we addressed was: should one expect as more likely that, when 16S sequences share 100% identity (‘clonal kins’) they: a) were recorded with the same taxonomic name; b) were isolated from closer rather than distant sites; c) were isolated from nodules of the same host plant ?”

Rhizobium is an important bacterial genus with implications for One Health, crop productivity, ecosystem nitrogen cycling, and sustainable agriculture, making this genus an intensively studied and important bacterial group for human enterprise. In addition, methods which utilize already existing data in publicly available databases are important contributions to the scientific community.

ANSWER: We thank Reviewer 3 for the extensive and thoughtful critique. Several of the issues raised overlap with concerns also noted by Reviewer 2, particularly regarding the need for a formal statistical framework, the interpretation of metadata concordance, and the degree of caution required in discussing the biological meaning of the results. In the revised manuscript, we have addressed these points by proposing a statistical analysis adapted to validate our observations, clarifying the scope of the study, improving the description of the methods, and moderating several formulations in the Introduction, Results, and Discussion.

In addition, we sincerely appreciate the correct understanding of the aims, including the fact that the Reviewer has caught the proper distinction of the “biological h-index” analogy. In fact, the concept stemmed from the data of the main analysis and appeared to us as worth reporting, also because, in spite of a scenario of little concordance of the records' environmental metadata, it indeed appears as a pure number that is independent of nomenclatural inconsistencies. We have added this suggested useful refined specification to its description.

========

Do the data and analyses fully support the claims? If not, what other evidence is required?

========

The analyses do not support the author's claims or question. The paper is only qualitative in nature, and no statistical framework is employed to test the author's hypothesis. This leaves the main questions (line 124) “should one expect as more likely that, when 16S sequences share 100% identity they (a) were recorded with the same taxonomic name, (b) were isolated from closer rather than distant sites (c) were isolated from nodules of the same host plant?” unanswered. Because the authors constrain the analysis only to sequences with 100% identity, they do not have the appropriate dataset to support statistical analysis of their proposed framework. For publication, the authors would need to develop and employ such a statistical framework.

In general, throughout the paper, only descriptive qualitative analyses are reported.

ANSWER: We thank the Reviewer for this important observation. We agree that a design restricted to 100% identity matches does not represent the full continuum of 16S divergence. However, we would like to underline that the objective of our study was not to model the entire gradient of sequence similarity, but rather to test the most stringent condition under which metadata concordance might reasonably be expected to be maximal, namely complete sequence identity combined with full query coverage across the aligned region. In this sense, the 100% identity criterion was used as a deliberate boundary condition for the analysis, not as an implicit claim that sequence identity alone is sufficient to establish species equivalence or full ecological similarity.

To address the Reviewer’s concern more rigorously, and as also requested by Reviewer 2, we re-evaluated the structure of our dataset and strengthened the analytical framework in the revised manuscript. We now distinguish a global full dataset (116069 matches) from two subsets: dataset B, comprising 52,048 full-length 100% identity matches with complete metadata, and dataset C, a more stringent subset of 11,422 matches in which species-level identification is explicit and unambiguous for both query and subject. The transition from B to C derives from a conservative anti-noise filtering strategy designed to exclude uninformative or taxonomically ambiguous entries, such as environmental clones, uncharacterized isolates, and generic strain designations, for example “Rhizobium sp.” records lacking unequivocal species resolution, or entries such as “Rhizobium sp. SEMIA 6437” and “Rhizobium sp. tg1”.

Within this revised framework, we believe the statistical analysis is no longer purely descriptive. We implemented a non-parametric null model based on 10,000 Monte Carlo permutations without replacement, preserving the marginal distribution of taxon, host, and country while randomizing the query-subject association. This allowed us to test directly whether the observed concordance patterns differ from random expectation within the background composition of the GenBank dataset itself. Under this model, several concordance classes in dataset B differed significantly from the null expectation, whereas the “same species only” class remained non-significant (p = 0.0817), which we consider informative because it reveals the contribution of unresolved or noisy taxonomic annotation in the broader dataset rather

Attachment Submitted filename: Answers to Reviewers.docx

# Peer review: Decision Letter 1

**DOI:** 10.1371/journal.pone.0357973.r003

**Authors:** Luigimaria Borruso

13 Aug 2026

Dear Dr. Squartini,

Please submit your revised manuscript by Sep 27 2026 11:59PM. If you will need more time than this to complete your revisions, please reply to this message or contact the journal office at plosone@plos.org. When you're ready to submit your revision, log on to https://www.editorialmanager.com/pone/ and select the 'Submissions Needing Revision' folder to locate your manuscript file.

- A letter that responds to each point raised by the academic editor and reviewer(s). You should upload this letter as a separate file labeled 'Response to Reviewers'.
- A marked-up copy of your manuscript that highlights changes made to the original version. You should upload this as a separate file labeled 'Revised Manuscript with Track Changes'.
- An unmarked version of your revised paper without tracked changes. You should upload this as a separate file labeled 'Manuscript'.

If you would like to make changes to your financial disclosure, please include your updated statement in your cover letter. Guidelines for resubmitting your figure files are available below the reviewer comments at the end of this letter.

If applicable, we recommend that you deposit your laboratory protocols in protocols.io to enhance the reproducibility of your results. Protocols.io assigns your protocol its own identifier (DOI) so that it can be cited independently in the future. For instructions see: https://journals.plos.org/plosone/s/submission-guidelines#loc-laboratory-protocols. Additionally, PLOS ONE offers an option for publishing peer-reviewed Lab Protocol articles, which describe protocols hosted on protocols.io. Read more information on sharing protocols at https://plos.org/protocols?utm_medium=editorial-email&utm_source=authorletters&utm_campaign=protocols.

As the corresponding author, your ORCID iD is verified in the submission system and will appear in the published article. PLOS supports the use of ORCID, and we encourage all coauthors to register for an ORCID iD and use it as well. Please encourage your coauthors to verify their ORCID iD within the submission system before final acceptance, as unverified ORCID iDs will not appear in the published article. *Only*  the individual author can complete the verification step; PLOS staff *cannot*  verify ORCID iDs on behalf of authors.

We look forward to receiving your revised manuscript.

Kind regards,

Luigimaria Borruso

Academic Editor

PLOS One

Journal Requirements:

If the reviewer comments include a recommendation to cite specific previously published works, please review and evaluate these publications to determine whether they are relevant and should be cited. There is no requirement to cite these works unless the editor has indicated otherwise.

Please review your reference list to ensure that it is complete and correct. If you have cited papers that have been retracted, please include the rationale for doing so in the manuscript text, or remove these references and replace them with relevant current references. Any changes to the reference list should be mentioned in the rebuttal letter that accompanies your revised manuscript. If you need to cite a retracted article, indicate the article’s retracted status in the References list and also include a citation and full reference for the retraction notice.

Additional Editor Comments :

Dear Andrea,

The revised manuscript has substantially improved, and the main concerns raised during the previous review have been addressed. Only a few minor revisions are required before acceptance.

Please replace expressions such as “ecological success”, “fitness”, “productivity”, and “pangenomic diversity” with more cautious wording, for example:

“a database-level metric of sequence recurrence and representation.”

These terms currently imply a biological meaning that is not directly demonstrated by sequence recurrence in GenBank and may also be affected by sampling and research effort.

- Abstract

Please replace:

“potential ecological prevalence, pangenomic diversity and spread”**

with, for example: “sequence recurrence, representation and distribution within public databases.”

In particular, “pangenomic diversity” is not directly assessed in the present study.

- Conclusions on 16S rRNA

Please replace:

**“increasingly weakening reliability of 16S-based species identification”**

with a more specific formulation such as:

**“limitations of relying on 16S rRNA alone for fine-scale taxonomic and metadata-based inference.”**

The present results support this more specific conclusion rather than a general statement on the overall reliability of 16S rRNA.

- Clonality terminology

Please replace “clonal kins” and “clonal relationships” with:

“sequences sharing 100% 16S identity”

or

“identical 16S sequences.”

Identical 16S sequences do not necessarily imply genome-wide clonality.

- Metadata standardization

Please replace:

“to eliminate taxonomic, botanical, and geographic assignment errors”

with:

“to reduce nomenclatural, formatting, and annotation inconsistencies.”

The standardization procedure can reduce inconsistencies in database records but cannot establish that the original biological assignments are correct.

[Note: HTML markup is below. Please do not edit.]

Reviewers' comments:

Reviewer's Responses to Questions

**Comments to the Author**

Reviewer #1: All comments have been addressed

Reviewer #2: All comments have been addressed

**********

2. Is the manuscript technically sound, and do the data support the conclusions?

Reviewer #1: Yes

Reviewer #2: Yes

**********

3. Has the statistical analysis been performed appropriately and rigorously?

Reviewer #1: Yes

Reviewer #2: Yes

**********

4. Have the authors made all data underlying the findings in their manuscript fully available?

The PLOS Data policy

Reviewer #1: Yes

Reviewer #2: Yes

**********

5. Is the manuscript presented in an intelligible fashion and written in standard English?

Reviewer #1: Yes

Reviewer #2: Yes

**********

Reviewer #1: The authors have satisfactorily addressed all of the reviewers’ comments. The revisions have significantly improved the scientific rigor, clarity, and overall quality of the manuscript. In my opinion, the manuscript is now suitable for publication and can be accepted in its current form.

Reviewer #2: The authors have adequately addressed the concerns raised during the previous round of review. The manuscript has been clearly improved, particularly with the addition of statistical analyses, better clarification of the methodology, and a more balanced interpretation of the findings and study limitations.

The authors have also provided detailed responses to the reviewers’ comments and made appropriate changes to the manuscript. I believe the revised version is scientifically stronger and the remaining limitations do not affect the main conclusions of the study.

The manuscript is suitable for publication in its current form.

**********

what does this mean?). If published, this will include your full peer review and any attached files.

If you choose “no”, your identity will remain anonymous but your review may still be made public.

**Do you want your identity to be public for this peer review?**  For information about this choice, including consent withdrawal, please see our Privacy Policy

Reviewer #1: No

Reviewer #2: No

**********

[NOTE: If reviewer comments were submitted as an attachment file, they will be attached to this email and accessible via the submission site. Please log into your account, locate the manuscript record, and check for the action link "View Attachments". If this link does not appear, there are no attachment files.]

To ensure your figures meet our technical requirements, please review our figure guidelines: https://journals.plos.org/plosone/s/figures

You may also use PLOS’s free figure tool, NAAS, to help you prepare publication quality figures: https://journals.plos.org/plosone/s/figures#loc-tools-for-figure-preparation.

NAAS will assess whether your figures meet our technical requirements by comparing each figure against our figure specifications.

# Author comment: Author response to Decision Letter 2

**DOI:** 10.1371/journal.pone.0357973.r004

17 Aug 2026

Additional Editor Comments :

Dear Andrea,

The revised manuscript has substantially improved, and the main concerns raised during the previous review have been addressed. Only a few minor revisions are required before acceptance.

Please replace expressions such as “ecological success”, “fitness”, “productivity”, and “pangenomic diversity” with more cautious wording, for example: “a database-level metric of sequence recurrence and representation.”

These terms currently imply a biological meaning that is not directly demonstrated by sequence recurrence in GenBank and may also be affected by sampling and research effort.

ANSWER: We agree, and we have changed the terminology as follows:

In substitution of the expression “ecological success” we have used the following: “a database-level metric of sequence recurrence and representation”; “recurrence proficiency”; “a given taxonomically-linked sequence representation”; “individual duplication and persistence proficiency”

In substitution of the word “fitness”: we have used the following: “individual genotype growth”; “adaptability”.

In substitution of the word “productivity”: we have used the term “reproductive potentiality”

The expression “pangenomic diversity” has been removed.

- Abstract

Please replace:

“potential ecological prevalence, pangenomic diversity and spread”**

with, for example: “sequence recurrence, representation and distribution within public databases.”

In particular, “pangenomic diversity” is not directly assessed in the present study.

ANSWER: The suggested substitution has been made.

- Conclusions on 16S rRNA

Please replace:

**“increasingly weakening reliability of 16S-based species identification”**

with a more specific formulation such as:

**“limitations of relying on 16S rRNA alone for fine-scale taxonomic and metadata-based inference.”**

The present results support this more specific conclusion rather than a general statement on the overall reliability of 16S rRNA.

ANSWER: The suggested substitution has been made.

- Clonality terminology

Please replace “clonal kins” and “clonal relationships” with:

“sequences sharing 100% 16S identity”

or

“identical 16S sequences.”

Identical 16S sequences do not necessarily imply genome-wide clonality.

ANSWER: The words “clonal” and “kins” have been removed

- Metadata standardization

Please replace:

“to eliminate taxonomic, botanical, and geographic assignment errors”

with:

“to reduce nomenclatural, formatting, and annotation inconsistencies.”

The standardization procedure can reduce inconsistencies in database records but cannot establish that the original biological assignments are correct.

ANSWER: The suggested substitution has been made.

Reviewers' comments:

Reviewer's Responses to Questions

Comments to the Author

1. If the authors have adequately addressed your comments raised in a previous round of review and you feel that this manuscript is now acceptable for publication, you may indicate that here to bypass the “Comments to the Author” section, enter your conflict of interest statement in the “Confidential to Editor” section, and submit your "Accept" recommendation.

Reviewer #1: All comments have been addressed

Reviewer #2: All comments have been addressed

________________________________________

2. Is the manuscript technically sound, and do the data support the conclusions?

The manuscript must describe a technically sound piece of scientific research with data that supports the conclusions. Experiments must have been conducted rigorously, with appropriate controls, replication, and sample sizes. The conclusions must be drawn appropriately based on the data presented.

Reviewer #1: Yes

Reviewer #2: Yes

________________________________________

3. Has the statistical analysis been performed appropriately and rigorously?

Reviewer #1: Yes

Reviewer #2: Yes

________________________________________

4. Have the authors made all data underlying the findings in their manuscript fully available?

The PLOS Data policy requires authors to make all data underlying the findings described in their manuscript fully available without restriction, with rare exception (please refer to the Data Availability Statement in the manuscript PDF file). The data should be provided as part of the manuscript or its supporting information, or deposited to a public repository. For example, in addition to summary statistics, the data points behind means, medians and variance measures should be available. If there are restrictions on publicly sharing data—e.g. participant privacy or use of data from a third party—those must be specified.

Reviewer #1: Yes

Reviewer #2: Yes

________________________________________

5. Is the manuscript presented in an intelligible fashion and written in standard English?

PLOS ONE does not copyedit accepted manuscripts, so the language in submitted articles must be clear, correct, and unambiguous. Any typographical or grammatical errors should be corrected at revision, so please note any specific errors here.

Reviewer #1: Yes

Reviewer #2: Yes

________________________________________

6. Review Comments to the Author

Please use the space provided to explain your answers to the questions above. You may also include additional comments for the author, including concerns about dual publication, research ethics, or publication ethics. (Please upload your review as an attachment if it exceeds 20,000 characters)

Reviewer #1: The authors have satisfactorily addressed all of the reviewers’ comments. The revisions have significantly improved the scientific rigor, clarity, and overall quality of the manuscript. In my opinion, the manuscript is now suitable for publication and can be accepted in its current form.

Reviewer #2: The authors have adequately addressed the concerns raised during the previous round of review. The manuscript has been clearly improved, particularly with the addition of statistical analyses, better clarification of the methodology, and a more balanced interpretation of the findings and study limitations.

The authors have also provided detailed responses to the reviewers’ comments and made appropriate changes to the manuscript. I believe the revised version is scientifically stronger and the remaining limitations do not affect the main conclusions of the study.

The manuscript is suitable for publication in its current form.

Hoping to have addressed all the pending issues, we thank you for your kind attention, and we look forward to hearing from you.

Attachment Submitted filename: Answers Second Round.docx

# Editor report: Decision Letter 2

**DOI:** 10.1371/journal.pone.0357973.r005

**Authors:** Luigimaria Borruso

25 Aug 2026

GenBank mining reveals novel insights into Rhizobium phylogeny: identical 16S rRNA sequences are mainly uncoupled from species designation, host plant, and geographic origin. How this search suggested the definition of a direct ‘microbial h-index’

PONE-D-26-01783R2

Dear Prof. Andrea Squartini,

We’re pleased to inform you that your manuscript has been judged scientifically suitable for publication and will be formally accepted for publication once it meets all outstanding technical requirements.

Within one week, you’ll receive an e-mail detailing the required amendments. When these have been addressed, you’ll receive a formal acceptance letter and your manuscript will be scheduled for publication.

An invoice will be generated when your article is formally accepted. Please note, if your institution has a publishing partnership with PLOS and your article meets the relevant criteria, all or part of your publication costs will be covered. Please make sure your user information is up-to-date by logging into Editorial Manager at Editorial Manager® and clicking the ‘Update My Information' link at the top of the page. For questions related to billing, please contact billing support.

If your institution or institutions have a press office, please notify them about your upcoming paper to help maximize its impact. If they’ll be preparing press materials, please inform our press team as soon as possible -- no later than 48 hours after receiving the formal acceptance. Your manuscript will remain under strict press embargo until 2 pm Eastern Time on the date of publication. For more information, please contact onepress@plos.org.

Kind regards,

Luigimaria Borruso

Academic Editor

PLOS One

Additional Editor Comments (optional):

Reviewers' comments:

# Editor report: Acceptance letter

**DOI:** 10.1371/journal.pone.0357973.r006

**Authors:** Luigimaria Borruso

PONE-D-26-01783R2

PLOS One

Dear Dr. Squartini,

I'm pleased to inform you that your manuscript has been deemed suitable for publication in PLOS One. Congratulations! Your manuscript is now being handed over to our production team.

At this stage, our production department will prepare your paper for publication. This includes ensuring the following:

* All references, tables, and figures are properly cited

* All relevant supporting information is included in the manuscript submission,

* There are no issues that prevent the paper from being properly typeset

You will receive further instructions from the production team, including instructions on how to review your proof when it is ready. Please keep in mind that we are working through a large volume of accepted articles, so please give us a few days to review your paper and let you know the next and final steps.

Lastly, if your institution or institutions have a press office, please let them know about your upcoming paper now to help maximize its impact. If they'll be preparing press materials, please inform our press team within the next 48 hours. Your manuscript will remain under strict press embargo until 2 pm Eastern Time on the date of publication. For more information, please contact onepress@plos.org.

You will receive an invoice from PLOS for your publication fee after your manuscript has reached the completed accept phase. If you receive an email requesting payment before acceptance or for any other service, this may be a phishing scheme. Learn how to identify phishing emails and protect your accounts at https://explore.plos.org/phishing.

If we can help with anything else, please email us at customercare@plos.org.

Thank you for submitting your work to PLOS One and supporting open access.

Kind regards,

PLOS One Editorial Office Staff

on behalf of

Dr. Luigimaria Borruso

Academic Editor

PLOS One
